# H_2_S generated by L-cysteine desulfhydrase (SlLCD1) enhances heat tolerance in tomato via antioxidant capacity and stomatal modulation

**DOI:** 10.1093/hr/uhag090

**Published:** 2026-03-09

**Authors:** Huihui Fang, Xiaofang Zhang, Yunfei Xu, Wenjia Chen, Kaixin Zheng, Weiling Zhao, Yijie Zang, Yunxiang Zang

**Affiliations:** Key Laboratory of Quality and Safety Control for Subtropical Fruit and Vegetable, Ministry of Agriculture and Rural Affairs, Key Laboratory of Vegetable Germplasm Innovation and Quality Breeding in the Province, College of Horticulture, Zhejiang A&F University, Hangzhou, Zhejiang, 311300, China; Key Laboratory of Quality and Safety Control for Subtropical Fruit and Vegetable, Ministry of Agriculture and Rural Affairs, Key Laboratory of Vegetable Germplasm Innovation and Quality Breeding in the Province, College of Horticulture, Zhejiang A&F University, Hangzhou, Zhejiang, 311300, China; Key Laboratory of Quality and Safety Control for Subtropical Fruit and Vegetable, Ministry of Agriculture and Rural Affairs, Key Laboratory of Vegetable Germplasm Innovation and Quality Breeding in the Province, College of Horticulture, Zhejiang A&F University, Hangzhou, Zhejiang, 311300, China; Key Laboratory of Quality and Safety Control for Subtropical Fruit and Vegetable, Ministry of Agriculture and Rural Affairs, Key Laboratory of Vegetable Germplasm Innovation and Quality Breeding in the Province, College of Horticulture, Zhejiang A&F University, Hangzhou, Zhejiang, 311300, China; Key Laboratory of Quality and Safety Control for Subtropical Fruit and Vegetable, Ministry of Agriculture and Rural Affairs, Key Laboratory of Vegetable Germplasm Innovation and Quality Breeding in the Province, College of Horticulture, Zhejiang A&F University, Hangzhou, Zhejiang, 311300, China; Key Laboratory of Quality and Safety Control for Subtropical Fruit and Vegetable, Ministry of Agriculture and Rural Affairs, Key Laboratory of Vegetable Germplasm Innovation and Quality Breeding in the Province, College of Horticulture, Zhejiang A&F University, Hangzhou, Zhejiang, 311300, China; Key Laboratory of Quality and Safety Control for Subtropical Fruit and Vegetable, Ministry of Agriculture and Rural Affairs, Key Laboratory of Vegetable Germplasm Innovation and Quality Breeding in the Province, College of Horticulture, Zhejiang A&F University, Hangzhou, Zhejiang, 311300, China; Key Laboratory of Quality and Safety Control for Subtropical Fruit and Vegetable, Ministry of Agriculture and Rural Affairs, Key Laboratory of Vegetable Germplasm Innovation and Quality Breeding in the Province, College of Horticulture, Zhejiang A&F University, Hangzhou, Zhejiang, 311300, China

## Abstract

Global warming is increasing the frequency of heat stress, a major abiotic constraint on crop growth and productivity. Hydrogen sulfide (H_2_S), a novel gasotransmitter, has been reported to enhance crops’ heat tolerance, yet its underlying mechanism remains poorly understood. Here, we provide genetic evidence confirming that L-cysteine desulfhydrase (SlLCD1, Solyc01g068160) was the enzymatic source of endogenous H_2_S in tomato heat adaptation. Dual activation of H_2_S signaling through both *SlLCD1* overexpression and exogenous application enhanced tomato heat tolerance. Conversely, CRISPR/Cas9-generated *SlLCD1* mutants (*cr-sllcd1*), deficient in heat-induced H_2_S production, displayed heightened heat sensitivity with accelerated wilting and increased oxidative damage, which was rescued by exogenous H_2_S application. Compared to wild-type plants, the mutants showed a compromised heat-induced increase in antioxidant enzyme activities and levels. This defect, along with the concomitant ROS accumulation and oxidative damage, was reversed by H_2_S pretreatment, underscoring the critical role of the SlLCD1-H_2_S module in maintaining ROS homeostasis during heat adaptation. Additionally, *cr-sllcd1* mutants exhibited attenuated heat-induced stomatal closure and increased stomatal density. H_2_S pretreatment rescued both of these defects, thereby optimizing the trade-off among transpirational cooling, water conservation, and photosynthetic efficiency. Overall, the SlLCD1-H_2_S module confers heat tolerance by a dual mechanism, coordinately enhancing antioxidant capacity and fine-tuning stomatal dynamics. Our study elucidates an important component of the H_2_S signaling pathway in plant heat tolerance and offers a promising tractable target for developing heat-tolerant tomato cultivars.

## Introduction

Global warming is profoundly affecting global food production and security, posing complex challenges to agricultural systems and food security worldwide. According to projections by the World Meteorological Organization (WMO), the global temperatures will be 1.1°C to 1.9°C above pre-industrial levels from 2024 to 2028 [1, 2]. The Sixth Assessment Report (AR6) produced by the Intergovernmental Panel on Climate Change (IPCC) underscored the severe threats posed by climate change to global food systems [3]. Findings show that each 1°C increase in global mean surface temperature (GMST) results in an annual global production loss of 5.5 × 10^14^ kcal, equating to a daily per capita deficit of 120 kcal (*P* < 0.001). Although progressive adaptation can partially mitigate these losses, substantial impacts are anticipated to persist. Consequently, it is imperative to screen and develop heat-tolerant crop varieties for agricultural production and food security [4]. As a major global vegetable, tomato (*Solanum lycopersicum* L.) is cultivated across varied climates but exhibits high sensitivity to high temperatures. By inducing hotter and drier transplanting conditions and a shorter phenological cycle, climate warming exacerbates the frequency of disease and pest outbreaks, leading to a significant reduction in tomato productivity [5, 6]. Therefore, elucidating the mechanisms of tomato responses to heat stress and screening heat-tolerant tomato varieties are of great significance.

Upon heat stress, reactive oxygen species (ROS), including superoxide anion (O_2_^−^), hydrogen peroxide (H_2_O_2_), and hydroxyl radical (-OH), rapidly accumulate in multiple cellular compartments. When this accumulation surpasses the plant’s antioxidant scavenging capacity, it tips the balance toward oxidative damage [7–9]. Therefore, to counteract heat stress, plants must precisely balance ROS production and scavenging to maintain cellular homeostasis and thus prevent oxidative damage [10–12]. Actually, a certain level of ROS accumulation acts as an important signaling molecule that activates plant responses and tolerance to heat stress [13, 14]. As the most stable ROS, H_2_O_2_ serves as a signaling molecule in early heat stress to activate heat shock factors (HSFs), which trigger rapid assembly of HSE-binding complexes and upregulates heat shock proteins (HSPs)/chaperones. This redox-sensitive regulatory network also extends to several transcription factors whose activities are modulated by redox changes [15, 16]. Moreover, ROS generated in chloroplasts function as plastid retrograde signals that communicate the redox status of key pools (e.g., plastoquinone and glutathione) to the nucleus, to enable the adjustment of gene expression [17–21]. Therefore, investigating ROS homeostasis and ROS signal transduction under heat stress is of great significance for elucidating plant heat tolerance mechanisms.

As key gatekeepers for gas exchange, stomata are intrinsically linked to both water conservation and photosynthetic efficiency, making their regulation a primary mechanism of heat avoidance response [22–26]. Numerous studies show that heat stress-induced changes in stomatal aperture and density vary across plant organs and species depending on the stress intensity and duration. Stomatal opening promotes evaporative cooling and enhances gas exchange, albeit at the expense of water retention. Consequently, compensatory regulation of stomatal density, aperture, and size represents a critical adaptive strategy through which plants optimize the trade-off between water conservation and thermal regulation [27–33]. ROS are well-established signaling molecules in stomatal closure, and their accumulation in the apoplast and chloroplasts is an early hallmark of this process [34–36]. ROS modulate stomatal aperture by participating in calcium and abscisic acid (ABA) signaling pathways, and concurrently, by directly regulating protein activity through oxidative post-translational modifications to fine-tune guard cell signaling [37–40]. However, the mechanisms by which plants integrate stomatal morphology with ROS metabolism and scavenging to confer heat tolerance merit further investigation, and the corresponding regulatory networks also await comprehensive elucidation.

Gasotransmitters are small gaseous molecules endogenously generated in living organisms that facilitate intercellular communication [41]. Combining the typical features of classical signaling molecules with the versatility and distinct properties of small molecules, gasotransmitters play a crucial role in various physiological processes [38, 42–50]. Hydrogen sulfide (H_2_S) was recognized as the third gasotransmitter following the earlier identification of nitric oxide (NO) and carbon monoxide (CO) [51]. In plants, H_2_S is primarily generated endogenously through the enzymatic degradation of cysteine (Cys). This process is mainly catalyzed by L-/D-Cys desulfhydrases (L/D-CDes), which facilitate the decomposition of L-Cys and D-Cys into ammonia, pyruvate, and H_2_S. As the principal substrate for H_2_S production, Cys has its synthesis and metabolism tightly coupled to H_2_S generation. O-Acetylserine (thiol) lyase (OASTL) catalyzes the terminal step in Cys biosynthesis, with certain isoforms also facilitating the reversible reaction that releases H_2_S as a byproduct during Cys formation [48, 52]. Notably, despite sharing sequence features with the OASTL family, L-cysteine desulfhydrase 1 (DES1) contains non-conserved amino acid substitutions in its β8A–β9A loop that impair its biosynthetic function [53]. Instead, DES1 exhibits closer functional homology to L-CDes enzymes, catalyzing L-Cys degradation for endogenous H_2_S production [53, 54]. Given the predominance of L-Cys in plants, the L-Cys-degrading enzymes are key contributors to endogenous H_2_S generation and are responsible for the majority of its production.

Emerging studies have demonstrated that H_2_S acts as an active participant of the intricate signaling network that regulates plant heat tolerance [48, 52, 55]. Foliar application of H_2_S donor enhanced wheat heat tolerance in a concentration-dependent manner [56]. Exogenous H_2_S treatment elevates the levels of antioxidants such as ascorbic acid, glutathione, flavonoids, and carotenoids in maize, thereby mitigating heat stress-induced oxidative damage [57]. H_2_S pretreatment induces HSPs (HSP70, HSP80, and HSP90) to enhance heat tolerance in strawberry [55]. H_2_S fine-tunes proline metabolic enzymes to drive its accumulation, thereby enhancing heat tolerance in tobacco suspension cells [58]. Furthermore, H_2_S improves heat tolerance in poplar by increasing the activity of S-nitrosoglutathione reductase (GSNOR) and reducing reactive oxygen/nitrogen damage [38, 48]. Ca^2+^-induced H_2_S accumulation, mediated by L-CDes activation, mitigates heat damage in tobacco suspension cells [59]. H_2_S acts downstream of NO and Heme/CO signaling to enhance heat stress response in tobacco and maize [60–62]. Collectively, H_2_S plays a crucial role in plant heat tolerance. However, most conclusions are based on pharmacological evidence, and with limited genetic support, the underlying signaling mechanisms remain largely unknown.

In this study, we provide genetic evidence using the *SlLCD1* overexpression lines (*OE-SlLCD1*) and the CRISPR/Cas9-generated *SlLCD1* mutants (*cr-sllcd1*) to confirm that H_2_S generated by SlLCD1 enhances heat tolerance in tomato. Our data demonstrated that the *cr-sllcd1* mutant lines exhibited markedly reduced heat tolerance compared to the wild type, and exogenous H_2_S application could rescue its heat-sensitive phenotype. Based on these findings, we aim to elucidate the mechanisms by which H_2_S enhances heat tolerance in tomato, focusing particularly on its coordinated regulation of ROS metabolism and stomatal morphology. The results are expected not only to advance our understanding of H_2_S signaling networks but also to provide theoretical support for breeding heat-resistant tomato varieties.

## Results

### SlLCD1-generated H_2_S contributes to the heat stress response in tomato.

**Figure 1:**
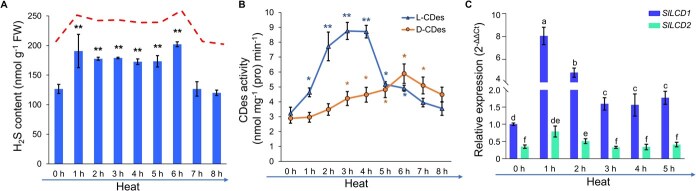
Dynamic response of the tomato endogenous H_2_S production system to heat stress. (A) Dynamic changes of endogenous H_2_S levels in WT tomato under heat stress. (B) Time-course of L-CDes and D-CDes activities in tomato during heat stress. (C) Expression dynamics of the H_2_S biosynthesis genes *SlLCD1* and *SlLCD2* in tomato under heat stress. The WT tomato plants at the six-true-leaf stage were exposed to 44°C, then the endogenous H_2_S content and the activities of L-CDes and D-CDes enzymes were measured at the indicated time points, 0, 1, 2, 4, 5, 6, 7, and 8 h after heat treatment, respectively. A time-course analysis of *SlLCD1* and *SlLCD2* expression levels was conducted up to 5 h. Asterisks denote statistical significance compared to the untreated control (0 h) (^*^p < 0.05 and ^**^p < 0.01), while different letters indicate significant differences between treatments (*p* < 0.05).

To investigate the response of endogenous H_2_S production to heat stress, we measured the endogenous H_2_S content and L/D-CDes enzymatic activities in tomato plants exposed to 44°C over a 0–8 h time course. Results showed that a biphasic response was observed in the endogenous H_2_S content, which significantly increased within the first 6 h of stress but subsequently returned to the baseline level within 7–8 h post stress (Fig. 1A). H_2_S declined to baseline in the later phase indicates the metabolic stabilization, reflecting the transient nature of signaling molecules that are rapidly attenuated once their functions are fulfilled. Correspondingly, the L-CDes activity responded rapidly to heat stress, increasing significantly within 2 h and surging to 2.71-fold over the baseline by 3 h (Fig. 1B), whereas D-CDes activity was also significantly induced but to a far less pronounced extent (Fig. 1B). In tomato, there are two LCD paralogs, one localized in the nucleus (SlLCD1, Solyc01g068160), another in the chloroplast (SlLCD2, Solyc05g007590) [50]. A comparative time-course analysis identified *SlLCD1* as the predominant isoform responding to heat stress. Its transcript level was rapidly and strongly induced, peaking at 1 h (Fig. 1C), and remained significantly and consistently higher than that of S*lLCD2* under both control and early stress conditions. In contrast, *SlLCD2* showed only a marginal induction at 1 h and 2 h, with its abundance and response magnitude remaining substantially lower (Fig. 1C). Other H_2_S-producing genes were also induced, but with slower and weaker responses than that of *SlLCD1* (Fig. S1). These results suggested that this early up-regulation of *SlLCD1*, coupled with the concomitant rise in L-CDes activity, drove the rapid accumulation of endogenous H_2_S and the activation of downstream signaling, underscoring the central role of SlLCD1-derived H_2_S in tomato heat adaptation.

### Dual activation of H_2_S signaling via *SlLCD1* overexpression and exogenous application enhances heat tolerance in tomato

**Figure 2:**
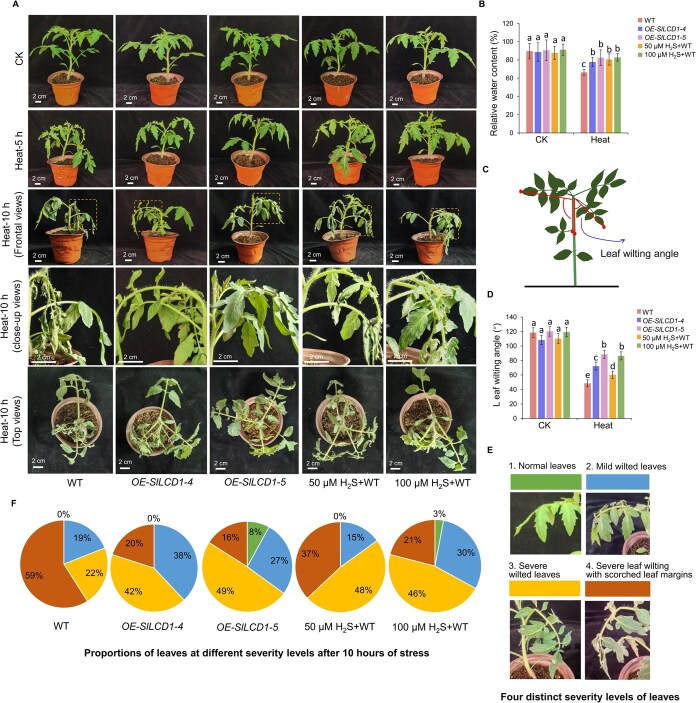
*SlLCD1* overexpression and exogenous H_2_S application enhance tomato heat tolerance. (A) Phenotypic characteristics of plants documented at specified time points during heat stress progression. Scale bar = 2 cm. (B) The leaf relative water content (RWC) of plants. (C) Schematic diagram showing the measurement of leaf wilting angle post-stress. (D) The leaf wilting angle of plants. (E) Schematic diagram showing four distinct severity levels of leaf conditions. (F) Proportions of leaves at different severity levels after 10 hours of stress. Uniformly grown plants of WT, *OE-SlLCD1* lines, and H_2_S-pretreated WT at the same growth stage (the six-true-leaf stage) were subjected to 44°C. The plant phenotypes were recorded (frontal and top views) at 0, 5, and 10 h after heat stress; the RWC of leaves and the leaf wilting angle were determined after 5 h of stress. The proportion of leaves at four distinct severity levels was quantified after 10 h of stress. For each treatment, the first six true leaves from six seedlings (36 leaves total) were assessed and categorized into four severity grades, with the percentage in each grade calculated relative to the total. Different letters represent statistically significant differences (*p* < 0.05).

To study the role of SlLCD1 in tomato heat adaptation, transgenic tomato lines overexpressing *SlLCD1* under the control of the CaMV 35S promoter (*OE-SlLCD1*) were generated. The successful overexpression of *SlLCD1*, as evidenced by its increased transcription in positive lines, elevated both the L-CDes activity and the endogenous H_2_S levels (Figs. S2A-D). During the early stage of heat stress, the *OE-SlLCD1* lines also exhibited higher L-CDes activity and H_2_S content compared to the WT, indicating a potentially enhanced H_2_S-mediated signaling response (Figs. S2E-F). Consistent with this, *OE-SlLCD1* lines displayed improved heat tolerance relative to the WT controls (Fig. 2A). Following 10 hours of heat exposure, WT leaves displayed pronounced wilting, curling, and margin scorching. In contrast, *OE-SlLCD1* plants showed only mild wilting and sustained better overall vitality under the same stress conditions (Fig. 2A). Correspondingly, after heat stress, the relative water contents (RWC) and leaf wilting angle of *OE-SlLCD1* lines were significantly superior to those of WT (Figs. 2B-D). To systematically characterize the physiological status of the plants, leaf damage was classified into four distinct grades (Fig. 2E). This quantification revealed that after 10 hours of heat stress, *OE-SlLCD1* lines showed a significantly higher proportion of moderately wilted leaves but a substantially lower proportion of severely wilted and scorched leaves compared to the WT (Figs. 2E-F). Remarkably, 8% of the leaves in the *OE-SlLCD1–5* line maintained a normal appearance (Figs. 2E-F). Collectively, these findings indicated that activating H_2_S signaling via *SlLCD1* overexpression improves heat tolerance in tomato.

A key characteristic of gaseous signaling molecules is that their physiological functions can be simulated by exogenous analogs, leading to the widespread pharmacological use of donors like NaHS in H_2_S research [41, 63]. Moreover, many H_2_S functions initially identified pharmacologically have been genetically validated, supporting the feasibility of probing H_2_S signaling through exogenous approaches. Based on a comprehensive review of the literature on exogenous H_2_S application (Table S1), two H_2_S concentrations (50 μM and 100 μM) were selected for pretreating tomato plants prior to heat stress. The results indicated that fumigation with both concentrations improved tomato heat tolerance to varying extents, as reflected by increased RWC, higher leaf wilting angles, and lower proportions of severely wilted and scorched leaves (Figs. 2A-F). Nevertheless, plants pretreated with 50 μM H_2_S showed a higher proportion of severely wilted and scorched leaves than those pretreated with 100 μM, indicating that 100 μM H_2_S fumigation is more effective in promoting thermotolerance (Figs. 2E-F). Therefore, this concentration was selected for complementation experiments to further establish the link between SlLCD1 function and H_2_S signaling during heat stress.

### H_2_S generation is weakened in *cr-sllcd1* mutant lines generated by CRISPR/Cas9

**Figure 3:**
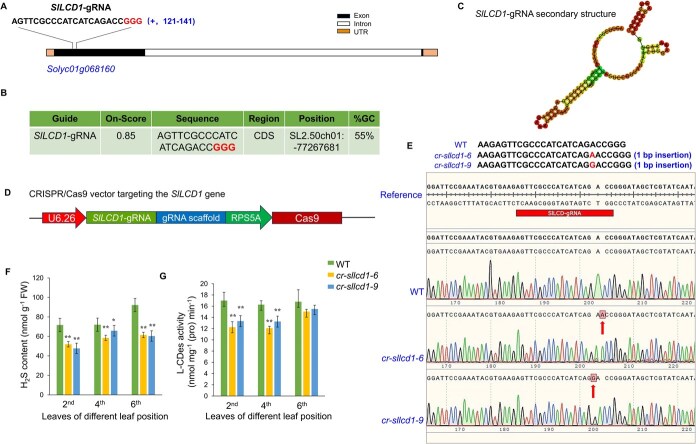
Characterization of gene-editing patterns and endogenous H_2_S production in *SlLCD1*-edited mutants. (A) Schematic representation of the *SlLCD1* gene and gRNA target site, with UTRs (orange), exons (black), introns (white), and the PAM sequence (red) indicated. (B) Details of *SlLCD1*-gRNA, On-score, target position, and GC content. (C) Predicted target-gRNA complex secondary structure. (D) CRISPR/Cas9 vector targeting the *SlLCD1* gene. (E) *SlLCD1*-editing patterns in the *cr-sllcd1–6* and *cr-sllcd1–9* mutant lines. Nucleotide insertion sites are highlighted with red arrows. (F and G) Comparison of the endogenous H_2_S levels (F) and L-CDes activity (G) across the 2^nd^, 4^th^, and 6^th^ nodal leaves of WT and *cr-sllcd1* mutants. Leaves of uniformly grown WT and *cr-sllcd1* mutant lines at the same growth stage (the six-true-leaf stage) were collected for the measurement of indicated parameters. Asterisks indicate significant differences compared to WT, ^*^p < 0.05 and ^**^p < 0.01.

To further investigate the function of SlLCD1-derived H_2_S in tomato heat adaptation, we generated CRISPR/Cas9-mediated *SlLCD1* edited mutants. A gRNA targeting the first exon of *SlLCD1* (AGTTCGCCCATCATCAGACC, PAM: GGG) (Figs. 3A-B) was designed and structurally predicted to form a loosely structured RNA conformation, a feature generally linked to higher targeting efficiency and accessibility (Fig. 3C). The gRNA was subsequently cloned into a CRISPR/Cas9 plasmid (Fig. 3D), and the resulting construct was introduced into the tomato genome via *Agrobacterium tumefaciens*-mediated transformation. Following PCR screening and editing pattern analysis, two transgenic lines, designated *cr-sllcd1–6* and *cr-sllcd1–9*, were confirmed to carry edited *SlLCD1* (Fig. 3E). These two mutant lines exhibited distinct insertion sites, and lines *cr-sllcd1–6* and *cr-sllcd1–9* carried single-base insertions 4 and 3 nucleotides upstream of the PAM site, respectively. Both lines carried frameshift mutations in *SlLCD1* that resulted in altered amino acid sequences and disrupted protein structures, confirming that *cr-sllcd1–6* and *cr-sllcd1–9* are successful gene-edited mutants (Fig. 3E). To predict potential off-target sites, we screened the tomato reference genome (SL2.50) using the CRISPR2 design tool [64] (http://crispr.hzau.edu.cn/CRISPR2/), allowing up to 4 mismatches with the *SlLCD1*-gRNA. Among the 24 predicted sites, the top 20 were intergenic, indicating low coding-region risk (Fig. S3A). PCR and Sanger sequencing of the top four sites in multiple homozygous mutant lines (*cr-sllcd1–6* and *cr-sllcd1–9*) revealed no insertions, deletions, or nucleotide variations relative to the WT (Ailsa Craig) (Fig. S3B). These results confirm the high editing specificity of the CRISPR system, thereby supporting the suitability of these lines as well-defined *SlLCD1* edited mutants.

We measured the endogenous H_2_S content and L-CDes activity in leaves at the 2^nd^, 4^th^, and 6^th^ leaf positions of the *cr-sllcd1–6* and *cr-sllcd1–9* mutants. Results showed that H_2_S levels at all examined leaf positions in both mutant lines were significantly lower than those in the WT (Fig. 3F). Specifically, the L-CDes activity was markedly reduced in the 2^nd^ and 4^th^ leaves of both mutants compared to the WT, while no significant difference was observed in the 6^th^ leaf (Fig. 3G). Given that L-CDes activity was measured via H_2_S generation from exogenous L-Cys, the detected signal could also arise from other L-Cys-degrading enzymes, such as SlLCD2, and the DES1 homologs SlOAS2 and SlOAS4 [65, 66]. Further RT-qPCR quantification revealed no significant change in *SlLCD2* expression, while both *SlOAS2* and *SlOAS4* were up-regulated in the 6^th^ leaf of the mutants (Fig. S3C), suggesting that mutation of *SlLCD1* may trigger a compensatory upregulation of other L-CDes members to restore cellular L-CDes activity (Fig. 3G). Yet total H_2_S levels in the mutant remained less than those of the WT (Fig. 3F), indicating either incomplete compensation or additional disruptions in H_2_S homeostasis. Future studies should employ systematic transcriptomic and enzymatic analyses to elucidate the full network of H_2_S synthesis and degradation during leaf development.

### SlLCD1-H_2_S module contributes to heat tolerance in tomato

**Figure 4:**
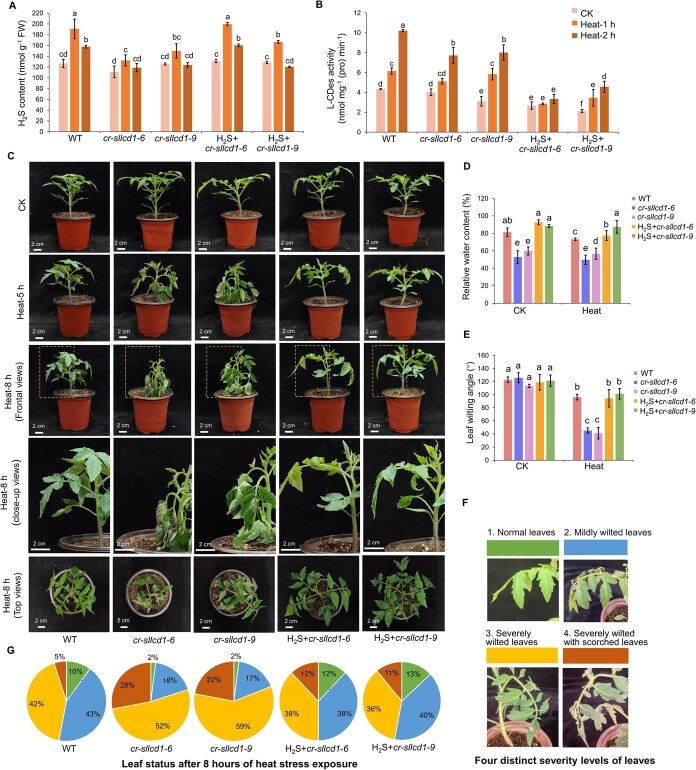
SlLCD1-H_2_S module positively enhances tomato heat tolerance. (A and B) Endogenous H_2_S content (A) and L-CDes activity (B) in WT, *cr-sllcd1* mutants, and H_2_S-pretreated *cr-sllcd1* mutants at indicated time points under heat stress. (C) Phenotypic characteristics of plants documented at specified time points during heat stress progression. Scale bar = 2 cm. (D and E) The leaf relative water content (RWC) (D) and the leaf wilting angle (E) of the corresponding plants under heat stress. (F) Schematic diagram showing four distinct severity levels of leaf conditions. (G) Proportions of leaves at different severity levels after 8 hours of stress. Uniformly grown plants of WT, *cr-sllcd1–6*, *cr-sllcd1–9*, H_2_S-pretreated mutants (designated H_2_S + *cr-sllcd1–6*, H_2_S + *cr-sllcd1–9*) at the same growth stage (the six-true-leaf stage) were subjected to 44°C. The endogenous H_2_S levels and L-CDes activity were assessed at 1 and 2 h post-treatment, and the plant phenotypes were recorded (frontal and top views) at 0, 5, and 8 h after heat stress. The leaf RWC and wilting angle were determined after 5 h of stress, and the proportion of leaves at four distinct severity levels was quantified after 8 h of stress. For each treatment, the first six true leaves from six seedlings (36 leaves total) were assessed and categorized into four severity grades, with the percentage in each grade calculated relative to the total. Different letters represent statistically significant differences (*p* < 0.05).

Consistent with the crucial role of SlLCD1 in heat-responsive H_2_S production, both endogenous H_2_S content and L-CDes activity were significantly upregulated in WT tomatoes after 1–2 h of heat stress, but this upregulation was strongly suppressed in *cr-sllcd1* mutants (Figs. 4A-B). Relative to the WT, *cr-sllcd1–6* and *cr-sllcd1–9* mutants exhibited significant reductions in the endogenous H_2_S content (by 24.61% and 21.33%, respectively) and the L-CDes activity (by 29.1% and 22.3%, respectively) following 2 h of heat stress (Figs. 4A-B). Further analysis revealed that *SlLCD1* mutation alters the heat stress responses of other H_2_S metabolic pathways. At the transcriptional level, the induction of *SlLCD2* and *SlOAS2* was stronger in the mutant at 2 h post-stress (Figs. S4A-B), while *SlOAS4* was activated more rapidly and strongly in the mutant, displaying significantly higher expression level as early as 1 h after heat stress (Fig. S4C). *SlDCD1* transcript levels were initially higher in the mutant at 2 h of heat stress, but the WT displayed substantially greater induction by 4 h (Fig. S4D). *SlDCD2* exhibited a similar temporal and quantitative induction pattern in both the WT and the *cr-sllcd1–6* mutant (Fig. S4E). In parallel, although D-CDes activity was initially higher in *cr-sllcd1* lines under normal conditions and at 1 h of heat stress, the marked activity increase observed in the WT after 2 h of heat stress was significantly attenuated in the mutant lines (Fig. S4F). These findings indicate that other H_2_S metabolic pathways may be enhanced in the mutant under heat stress, but they cannot fully compensate for the pronounced reduction in overall L-CDes activity and H_2_S levels. Consequently, the mutants remain functionally impaired in H_2_S signaling activation during heat stress.

Further analysis confirmed the critical role of *SlLCD1* in heat tolerance, as its mutation resulted in enhanced heat sensitivity in tomato. Following 5 h of heat treatment, leaves of both *cr-sllcd1–6* and *cr-sllcd1–9* mutants exhibited pronounced wilting and drooping, whereas those of the WT remained turgid and healthy (Fig. 4C). After 8 h of heat exposure, WT plants also began to show visible wilting, while the mutants developed more severe symptoms, including scorched and dehydrated leaf margins (Fig. 4C). To further confirm that the heat-sensitive phenotype of *cr-sllcd1* mutants was a consequence of reduced H_2_S levels, we examined the effects of exogenous H_2_S pretreatment on the performance of the *cr-sllcd1* mutants under heat stress. Notably, H_2_S pre-application alleviated heat-induced damage in the mutants and rescued their heat-sensitive phenotypes (Fig. 4C). Detached leaves from stressed *cr-sllcd1* mutants exhibited a higher water loss rate, a phenotype that was rescued by H_2_S application (Fig. S4G). Correspondingly, the RWC and leaf wilting angle of both *cr-sllcd1–6* and *cr-sllcd1–9* mutants were significantly lower than that of the WT, whereas mutants pretreated with exogenous H_2_S (H_2_S + *cr-sllcd1–6* and H_2_S + *cr-sllcd1–9*) showed levels of both indicators comparable to the WT (Figs. 4D-E). Quantification of severity levels after 8 hours of heat stress revealed that, compared to the WT, the *cr-sllcd1* mutants had a significantly lower proportion of mildly wilted leaves but a substantially higher proportion of severely wilted/scorched leaves (Figs. 4F-G). Impressively, H_2_S pretreatment demonstrated a clear rescuing effect, restoring the proportion of mildly affected leaves in mutants to near-WT levels and markedly reducing the severely wilted/scorched leaves, although full recovery was not achieved (Figs. 4F-G). Collectively, these findings indicate that H_2_S derived from SlLCD1 is essential for tomato water conservation under heat stress, and thus for subsequent heat adaptation.

### SlLCD1-H_2_S module functions in alleviating heat stress caused oxidative damage

**Figure 5:**
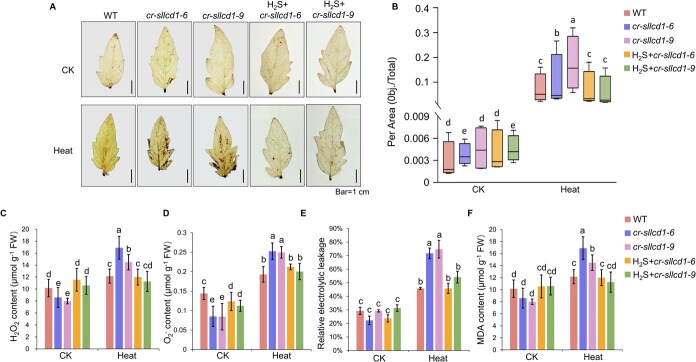
SlLCD1-H_2_S module modulates ROS accumulation and alleviates oxidative damage in tomato under heat stress. (A) Phenotype analysis of DAB staining in leaves of WT, *cr-sllcd1* mutants, and H_2_S-pretreated *cr-sllcd1* mutants under heat stress. The intensity of brown coloration reflects the level of H_2_O_2_ accumulation. Scale bar = 1 cm. (B) ImageJ analysis of DAB staining intensity shown in (A). (C–F) Contents of H_2_O_2_, superoxide anion (O_2_^−^), relative electrolytic leakage (REL), and malondialdehyde (MDA) in tomato leaves under heat stress. Uniformly grown plants of WT, *cr-sllcd1–6*, *cr-sllcd1–9*, H_2_S-pretreated mutants (designated H_2_S + *cr-sllcd1–6*, H_2_S + *cr-sllcd1–9*) at the same growth stage (the six-true-leaf stage) were subjected to 44°C. The indicated parameters were measured at 0 h (CK) and 5 h (Heat) after heat stress. Different lowercase letters indicate statistically significant differences (*p* < 0.05).

Maintaining ROS metabolic homeostasis is critical for the stability of the cell membrane system under heat stress. DAB staining revealed deeper brown pigmentation, indicative of higher H_2_O_2_ accumulation, in the leaves of *cr-sllcd1* mutants compared to the WT under heat stress (Fig. 5A). In line with this visual observation, quantitative analysis of mean pixel intensity using ImageJ confirmed higher DAB staining in the mutants after heat stress (Fig. 5B). Conversely, exogenous H_2_S treatment effectively reduced this staining intensity in the mutants, which was supported by both visual and quantitative data (Figs. 5A-B). Spectrophotometric quantification confirmed a significant heat-induced accumulation of H_2_O_2_ and O_2_^−^, which was more pronounced in the *cr-sllcd1* mutants. Whereas exogenous H_2_S supplementation led to a marked attenuation of ROS accumulation in these mutant lines (Figs. 5C-D). Consistent with increased oxidative damage, heat stress significantly elevated relative electrolyte leakage (REL) and malondialdehyde (MDA) content, a characteristic end-product of lipid peroxidation, with this effect being more pronounced in the *cr-sllcd1* mutants. Following 5 hours of heat stress, *cr-sllcd1–6* and *cr-sllcd1–9* exhibited 55.3% and 63.8% higher REL levels, and 36.4% and 14.3% higher MDA contents, respectively, compared to WT (Figs. 5E-F). H_2_S pretreatment restored both oxidative damage indicators in the mutant to WT levels (Figs. 5E-F). These results provide genetic evidence that H_2_S generated by SlLCD1 reduces excessive ROS accumulation under heat stress and alleviates oxidative damage, which may represent an important mechanism through which H_2_S enhances heat tolerance in tomato.

### SlLCD1-H_2_S module enhances activities of antioxidant enzymes under heat stress

**Figure 6:**
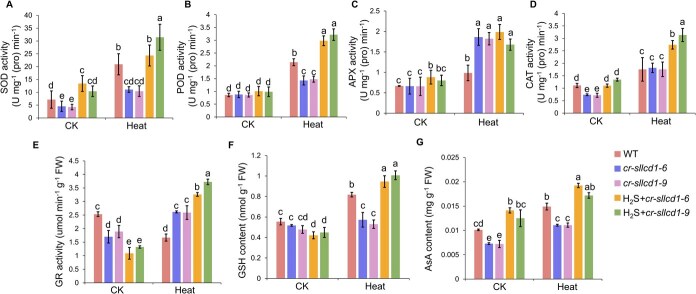
SlLCD1-H_2_S module enhances the antioxidant capacity in tomato under heat stress. (A-E) Activities of the antioxidant enzymes SOD, POD, APX, CAT, and GR in leaves of WT, *cr-sllcd1* mutants, and H_2_S-pretreated *cr-sllcd1* mutants following heat stress. (F-G) Contents of reduced GSH and ASA in tomato under heat stress. Uniformly grown plants of WT, *cr-sllcd1–6*, *cr-sllcd1–9*, H_2_S-pretreated mutants (designated H_2_S + *cr-sllcd1–6*, H_2_S + *cr-sllcd1–9*) at the same growth stage (the six-true-leaf stage) were subjected to 44°C. The indicated parameters were measured at 0 h (CK) and 5 h (Heat) after stress. Different lowercase letters indicate statistically significant differences (*p* < 0.05).

To investigate the mechanism by which SlLCD1-H_2_S module alleviates heat stress-induced oxidative damage, we assessed the antioxidant system in WT, *cr-sllcd1* mutants, and H_2_S-pretreated mutants (H_2_S + *cr-sllcd1*) after heat stress. The results demonstrated that heat stress significantly enhanced the activities of superoxide dismutase (SOD), peroxidase (POD), ascorbate peroxidase (APX), and catalase (CAT) in both WT and *cr-sllcd1* mutants (Figs. 6A-D). However, compared to the WT, the upregulation of both SOD and POD activities was significantly attenuated in *cr-sllcd1–6* and *cr-sllcd1–9*, while APX activities exhibited a more pronounced increase in the mutants (Figs. 6A-C). The CAT activity showed no significant difference between the WT and the mutants following heat stress (Fig. 6D). In contrast, heat stress significantly reduced the glutathione reductase (GR) activity in the WT, but markedly increased it in *cr-sllcd1* mutants (Fig. 6E). This opposing regulatory pattern suggested that the SlLCD1-H_2_S module normally fine-tunes redox balance, prioritizing direct scavenging over the metabolically costly GR pathway to conserve NAD(P)H. The upregulation of GR activity in *cr-sllcd1* mutant is likely a compensatory response to the primary antioxidant deficiency and a severe ROS burst (Fig. 5, Fig. 6). Nevertheless, this response may be self-limiting, as the accompanying ROS surge could impair NAD(P)H regeneration, thereby constraining GR efficacy. In addition, heat stress induced the accumulation of key non-enzymatic antioxidants, reduced glutathione (GSH) and ascorbic acid (AsA), in all genotypes, albeit to a lesser extent in the *cr-sllcd1–6* and *cr-sllcd1–9* compared to the WT (Figs. 6F-G). Notably, exogenous H_2_S treatment enhanced the activities of these antioxidant enzymes in both *cr-sllcd1–6* and *cr-sllcd1–9*, reaching levels that significantly exceeded those in the WT (Figs. 6A-E). These results indicated that exogenous H_2_S treatment induced a more comprehensive activation of the antioxidant system. This enhanced response likely stems either from the pre-emptive application of H_2_S, enabling faster signal transduction, or from a reduced baseline sensitivity of these enzymes to H_2_S in *cr*-*sllcd1* mutants, which permits a greater activating effect from exogenous treatment. Together, plants activate a coordinated antioxidant response to mitigate heat-induced ROS bursts, and this fine-tuning process is disrupted by *SlLCD1* mutation, suggesting that the activation and orchestration of antioxidant capacity in plants depends, at least partially, on the SlLCD1-H_2_S module.

### SlLCD1-H_2_S module orchestrates stomatal density and aperture under heat stress

**Figure 7:**
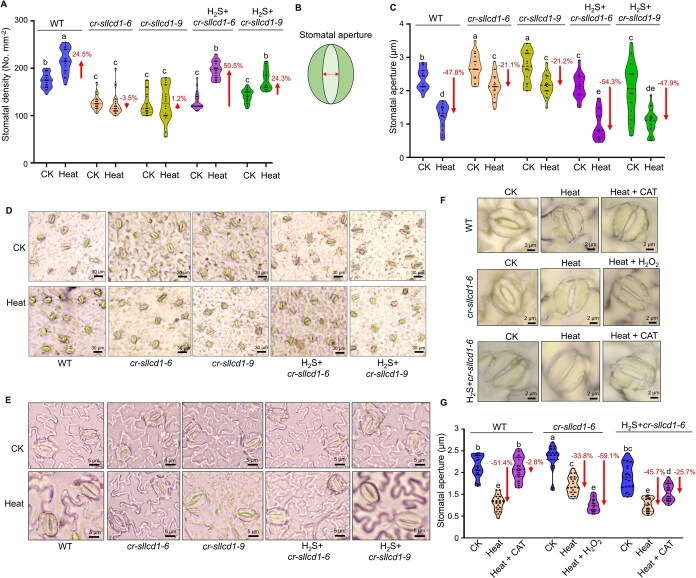
SlLCD1-H_2_S module regulates the stomatal density and aperture during heat stress. (A and D) The phenotypic and statistical analysis of stomatal density in leaves of WT, *cr-sllcd1* mutants, and H_2_S-pretreated *cr-sllcd1* mutants under heat stress. Stomatal density in the newly developed leaves of the indicated plants was analyzed at two time points, at 0 h (CK) and after a 24 h recovery period following 5 hours of heat stress (Heat). (B) Diagram for stomatal aperture measurement, and the aperture was quantified as the maximum pore width between the guard cells. (C and E) The phenotypic and statistical analysis of stomatal aperture in leaves of WT, *cr-sllcd1* mutants, and H_2_S-pretreated *cr-sllcd1* mutants under heat stress. Uniformly grown plants of WT, *cr-sllcd1–6*, *cr-sllcd1–9*, H_2_S-pretreated mutants (designated H_2_S + *cr-sllcd1–6*, H_2_S + *cr-sllcd1–9*) at the same growth stage (the six-true-leaf stage) were subjected to 44°C heat stress. Stomatal aperture in the fully expanded leaves of the indicated plants was analyzed 0 h (CK) and 5 h (Heat) after heat stress. (F and G) The phenotypic and statistical analysis of stomatal aperture in isolated leaf epidermal strips from WT, *cr-sllcd1* mutants, and H_2_S-pretreated *cr-sllcd1* mutants under heat stress, either in the absence or presence of 1 mM H_2_O_2_ or 100 U/L CAT. The sampling protocol consisted of three biological replicates per treatment, with three distinct fields of view examined for every leaf. Within every field, both the number of all stomata and the aperture size of at least 20 stomata were measured using ImageJ software. The red indicator lines/values in A, C, and G indicate the percentage of increase (upregulation) or decrease (downregulation) in stomatal aperture/density under heat stress compared to the untreated control. The Different letters indicate statistically significant differences (*p* < 0.05).

Our investigation revealed that WT and *cr-sllcd1* mutants exhibited pronounced phenotypic divergence in stomatal morphology, specifically in density and aperture, under heat stress (Fig. 7). Because stomatal development is a long-term process, we measured the stomatal density of newly developed leaves after a 5-h heat stress followed by a 24-h recovery period. Data indicated that heat stress significantly increased the stomatal density of newly developed leaves in WT plants, whereas this response was completely abolished in the *cr-sllcd1* mutants, leaving stomatal density no significant difference from their respective controls (Figs. 7A, 7D). The leaves of both *cr-sllcd1–6* and *cr-sllcd1–9* mutants exhibited a significantly lower stomatal density than the WT, with reductions of 43.2% and 21.7%, respectively, following stress exposure. H_2_S pretreatment partially restored the heat-induced increase of stomatal density in the *cr-sllcd1* mutants, while the final stomatal density in H_2_S + *cr-sllcd1* remained lower than in the WT (Figs. 7A, 7D). These results suggest that the SlLCD1-H_2_S module participates in promoting stomatal development and increasing stomatal density under heat stress, potentially enhancing cooling via increased transpiration.

Heat stress reduced stomatal aperture, defined as the maximum width between the guard cells (Fig. 7B), in all lines examined, WT, *cr-sllcd1* mutants, and H_2_S-pretreated *cr-sllcd1* mutants (Figs. 7C, 7E). However, this reduction was less pronounced in the mutants than in the WT, indicating an attenuated heat-induced stomatal closure caused by *SlLCD1* mutation (Figs. 7C, 7E). Notably, this attenuation of stomatal closure in mutants was fully reversed by H_2_S pretreatment. Following heat stress, leaves of H_2_S-pretreated *cr-sllcd1* mutants (H_2_S + *cr-sllcd1*) exhibited the smallest stomatal aperture among all genotypes (Figs. 7C, 7E). Collectively, the SlLCD1-H_2_S module may coordinately modulate both stomatal density and aperture, fine-tuning the balance between transpirational cooling and water retention during heat stress, as evidenced by H_2_S’s ability to rescue the heat-wilting phenotype and restore RWC in the *cr-sllcd1–6* and *cr-sllcd1–9* mutants (Figs. 4C-G).

Combining the established function of H_2_O_2_ signaling in stomatal regulation with our prior finding that SlLCD1-H_2_S modulates ROS homeostasis under heat stress, we next tested whether H_2_O_2_ mediates H_2_S-induced stomatal closure using epidermal strip assays with exogenous H_2_O_2_ and its scavenger CAT. Data indicated that the *cr-sllcd1–6* mutant exhibited significantly reduced stomatal closure under heat stress compared to the WT, and this impairment was effectively rescued by exogenous H_2_O_2_ application (Fig. 7F-G). H_2_S treatment also significantly alleviated the heat-induced stomatal closure defect in the mutant, although it did not restore closure to the WT level (Fig. 7F-G). Notably, when co-treated with the CAT under heat stress, the restorative effect of H_2_S was completely abolished (Fig. 7F-G). These results collectively indicate that H_2_O_2_ signaling likely functions downstream of SlLCD1-H_2_S in mediating stomatal closure during heat stress, though the precise mechanistic relationship warrants further investigation.

### SlLCD1-H_2_S module enhances photosynthetic intensity of tomato under heat stress.

**Figure 8:**
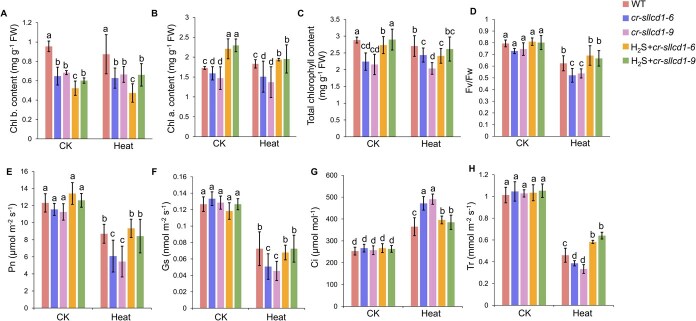
SlLCD1-H_2_S module enhances the photosynthetic efficiency during heat stress. (A-C) The contents of photosynthetic pigments, Chl. b, Chl. a, and total Chl., in WT, *cr-sllcd1* mutants, and H_2_S-pretreated *cr-sllcd1* mutants under heat stress. (D-H) The maximum quantum efficiency of PSII photochemistry (Fv/Fm), net photosynthesis rate (Pn), intercellular CO_2_ concentration (Ci), stomatal conductance (Gs), and transpiration rate (Tr) in leaves of WT, *cr-sllcd1* mutants, and H_2_S-pretreated *cr-sllcd1* mutants under heat stress. Uniformly grown plants of WT, *cr-sllcd1–6*, *cr-sllcd1–9*, H_2_S-pretreated mutants (designated H_2_S + *cr-sllcd1–6*, H_2_S + *cr-sllcd1–9*) at the same growth stage (the six-true-leaf stage) were subjected to 44°C. The indicated parameters were measured at 0 h (CK) and 5 h (Heat) after heat stress. Different lowercase letters indicate statistically significant differences (*p* < 0.05).

Our data indicated that under both normal and heat-stress conditions, the Chl. b content in both mutants and the H_2_S-pretreated mutants were consistently lower than that in WT plants (Fig. 8A). Regardless of stress exposure, the Chl. a and total Chl. contents in *cr-sllcd1–6* and *cr-sllcd1–9* were lower than those in the WT, while exogenous application of H_2_S compensated for this deficit (Figs. 8B-C). In addition, the ‘super-compensation’ phenomenon, wherein Chl. a content in the H_2_S-pretreated mutants exceeded those in the WT (Fig. 8B), represents a nonlinear recovery process. We propose that this phenomenon may arise result from multiple, non-exclusive mechanisms. The enhanced sensitivity of key chlorophyll synthesis enzymes to H_2_S in the mutants may enable exogenous H_2_S to hyper-activate their activity, temporarily elevating Chl. a synthesis above WT levels. Alternatively, underlying photosynthetic defects may exist in the mutants, which trigger H_2_S-induced compensatory overproduction of Chl. a to optimize light capture. Future studies comparing the persulfidation profiles, chlorophyll-biosynthesis intermediates, and transcriptomic responses between H_2_S-treated mutants and the WT could facilitate the differentiation of these possibilities.

Compared to the WT, the *cr-sllcd1* mutants suffered more severe PSII photoinhibition under heat stress, as reflected by a significant decrease in the maximum quantum efficiency of PSII photochemistry (Fv/Fm) (Fig. 8D). This confirmed that SlLCD1-H_2_S module acts importantly in protecting PSII from heat damage. More importantly, exogenous H_2_S pretreatment fully restored the mutant’s Fv/Fm to a WT-comparable level (Fig. 8D). In addition, the photosynthesis related key parameters such as net photosynthesis rate (Pn), stomatal conductance (Gs), intercellular CO_2_ concentration (Ci) and transpiration rate (Tr) were also measured to assess the photosynthetic efficiency. Although no genotypic or treatment differences in Pn, Gs, Tr, and Ci were detected under normal conditions, heat stress triggered a consistent response in WT and *cr-sllcd1* mutants, a significant decrease in Pn, Gs, and Tr coupled with an increase in Ci (Figs. 8E-H). Heat stress-induced reductions in Pn, Gs, and Tr were more pronounced in the *cr-sllcd1–6* and *cr-sllcd1–9* mutants, and H_2_S pretreatment alleviated these declines, restoring these parameters to at least WT levels, with Tr being even beyond that of the WT (Figs. 8E-F, 8H). Additionally, H_2_S pretreatment partially restored the heat stress-induced hyper-upregulation of Ci in the *cr-sllcd1* mutants to an intermediate level between the WT and the mutants (Fig. 8G). In *cr-sllcd1* mutants, the parallel decrease in Tr and RWC (Figs. 4D, 8H) exposes a severe breakdown in the trade-off between transpirational cooling and water conservation. The reversal of this deleterious phenotype by H_2_S definitively establishes the SlLCD1-H_2_S module as the key regulatory hub for this essential balance. Together, SlLCD1-H_2_S module as a critical signaling hub orchestrating photosynthetic responses to heat stress. It not only protects PSII but also functions as a key regulatory mechanism for maintaining the thermal stability of the photosynthetic apparatus in plants. This function is likely associated with its regulation of stomatal morphology, which governs transpiration and CO_2_ uptake.

## Discussion

Following the initial discovery of leaf H_2_S release in plants such as cucumber, corn, and soybean in 1978 [67], CDes catalyzed Cys degradation has been identified as the primary pathway for endogenous H_2_S production in plants [68–71]. LCD, a key L-CDes enzyme, is critical for H_2_S production in plants, which makes *lcd* mutants an essential genetic tool for dissecting H_2_S signaling in modern studies. Genetic evidence from *LCD* mutants has established that LCD, via H_2_S generation and signaling, serves as a central hub underpinning antioxidant capacity, stomatal development and movement, and diverse physiological processes, including seed germination, fruit ripening, and tolerance to drought, Cr^6+^, and Cd^2+^ stress, in plants such as *Arabidopsis*, tomato, and rice [50, 72–79]. By demonstrating that CRISPR/Cas9-generated *SlLCD1* mutants (*cr-sllcd1*) displayed heightened heat sensitivity and H_2_S pretreatment restores heat tolerance in these mutants (Fig. 4C-G), our study genetically corroborates previous pharmacological evidence for H_2_S enhanced heat tolerance in plants and further implicates SlLCD1 functioning in the modulation of redox homeostasis and stomatal morphology under heat stress.

Based on our data, endogenous H_2_S levels showed a transient increase under heat stress, rising initially and then returning to baseline (Fig. 1A). Our primary study in MicroTom tomato under moderate stress (37°C) confirmed this dynamic pattern of endogenous H_2_S, a rapid increase followed by a gradual decrease [65]. The transient kinetics of H_2_S reflect its function as a signaling molecule that requires rapid inactivation after signaling. In plants, this is accomplished by dedicated clearance pathways, primarily through mitochondrial oxidation (via sulfide:quinone oxidoreductase (SQR) and/or sulfur dioxygenase (SDO)) or OASTL-mediated assimilation, illustrating a ‘signaling–metabolism synergy’ [80, 81]. This process not only terminates the signal but also channels the released sulfur into metabolism, producing cysteine and other organic compounds. Concurrently, enzymatic H_2_S production returns to baseline levels (Fig. 1A), ensuring signal specificity, minimizing toxicity, and optimizing sulfur use efficiency, thereby supporting adaptation to environmental changes. Specifically, under moderate stress (37°C), the H_2_S response was notably sustained, levels began to decrease after 24 h but remained above baseline even at 60 h [65]. In contrast, extreme heat (44°C) induced only a transient H_2_S dynamic, demonstrating a specific stress-intensity-dependent response. Moving forward, it will be essential to systematically elucidate how H_2_S signaling diverges under moderate versus extreme heat stress, and to identify the key biosynthetic enzymes that are differentially regulated in each scenario. Several testable hypotheses arise from these observations. Firstly, the differential H_2_S accumulation dynamics across stress intensities may stem from transcriptional or post-translational regulation of specific enzymes. Although the chloroplast-localized SlLCD2 shows a relatively weak transcriptional response to heat stress compared to SlLCD1 (Fig. 1C), it remains essential to genetically dissect the function and underlying mechanism of SlLCD2 in tomato heat adaptation, particularly in chloroplast-related processes. CRISPR/Cas9-generated knockout lines would help clarify whether this isoform contributes to thermotolerance via chloroplast retrograde signaling or modulation of photosynthetic efficiency, thereby completing the picture of LCD-mediated H_2_S signaling under heat stress. As another key member of L-CDes, DES1 plays a significant role in endogenous H_2_S production, and our previous study based on the T-DNA insertion mutant of *DES1* confirms the role of H_2_S signaling in basal thermotolerance in Arabidopsis [82]. Therefore, it remains to be elucidated whether functional redundancy exists between SlLCD1 and SlDES1 in enhancing tomato heat tolerance, and whether their responses vary with the intensity and duration of heat stress. Secondly, the sustained H_2_S elevation observed under moderate heat stress could imply the involvement of HSF-mediated transcriptional control, a process likely tuned by stress intensity [83–85]. Moreover, elucidating the upstream regulators of stress-intensity-dependent H_2_S production offers a pivotal opportunity to advance H_2_S signaling research. Thirdly, the heat-tolerance-enhancing effect of H_2_S in tomato, achieved through its modulation of antioxidant systems and stomatal responses (as identified here), may vary depending on the intensity of heat stress. Deeper exploration of these regulatory pathways will clarify how H_2_S homeostasis underpins acclimation to moderate heat and may inform strategies to improve crop resilience in fluctuating thermal environments.

The *cr-sllcd1* mutants, generated in our present study, exhibited increased stomatal aperture compared to the WT (Figs. 7C, 7E), aligning with the widely reported function of H_2_S in inducing stomatal closure (particularly under drought conditions) [37, 39, 44, 47, 49, 75, 86–88]. The reported targets and mechanisms underlying H_2_S-regulated stomatal closure can be classified into three levels: 1) Transcriptional regulation, through modulation of key ion channel gene expression, including *AKT1*/*AKT2*/*KAT1*/*KC1* for K^+^ influx, *GORK*/*SKOR*/*KCO1* for K^+^ efflux, Ca^2+^ channels *TPC1*, and anion channels *SLAC1* [76]; 2) Post-translational modification, via the persulfidation of ion channels such as KAT1 (at Cys647) [47], AKT1 and KCO1 [88]; critical proteins such as the kinase OST1 (at Cys131/137) [89] and RBOHD (at Cys825/890) [87]; succinate dehydrogenase SDH1–1 [90, 91]; and 3) Signaling crosstalk, involving integrative interactions with pathways mediated by H_2_O_2_, ABA, JA, Ca^2+^, NO, and CO [37, 38, 92–95]. Here, we found that the impairment of heat-induced stomatal closure in *cr-sllcd1* mutants was restored by exogenous H_2_S application (Figs. 7C, 7E), extending the well-established role of H_2_S in stomatal closure from conferring drought tolerance to a broader function in heat adaptation. This raises the question of whether H_2_S-induced stomatal closure under heat stress operates through these reported mechanisms, with the precise mechanism and its potential interactions with other signaling pathways remaining to be systematically elucidated.

Beyond triggering rapid stomatal closure, H_2_S has also been implicated in long-term stomatal development [93, 96, 97]. Based on our study, *cr-sllcd1* had significantly decreased stomatal density in their new leaves compared to WT, supporting the function of H_2_S in positively regulatiing stomatal development. Critically, H_2_S pretreatment restored the defective heat-induced stomatal density increase in mutants, providing direct evidence that the SlLCD1-H_2_S module is necessary for modulating stomatal development under heat stress. This observation aligns with our earlier findings in Arabidopsis, where H_2_S signaling was shown to coordinate both stomatal aperture and density, thereby contributing to basal thermotolerance [82]. Similarly, Bo et al. reported that H_2_S promotes stomatal development in emerging leaves through persulfidation of EPF2/EPFL9 [96]. However, existing reports on H_2_S-mediated stomatal development present a complex picture. A study by Deng et al. reported an opposing role for H_2_S, demonstrating that it functions as an inhibitory signal for stomatal density in the MeJA pathway within cotyledons [93]. The seemingly contradictory roles of H_2_S in stomatal development are, in fact, a reflection of its multifaceted and context-dependent nature as a signaling molecule. To integrate these findings, we propose a model in which the effects of H_2_S depend on the interplay of developmental stage, spatial signaling, and environmental context. Firstly, H_2_S may play a dynamic, biphasic role in stomatal development, shifting from promoting lineage proliferation and early differentiation to suppressing division and enforcing terminal differentiation. Future comparative analysis of stomatal lineage progression in H_2_S-deficient mutants versus WT plants is essential to dissect how H_2_S fine-tunes this proliferation-differentiation switch. Secondly, H_2_S likely integrates systemic cues, notably mesophyll-derived ABA, to translate environmental stress into localized stomatal adaptation. Thirdly, under stress, H_2_S might serve as a key node that tunes stomatal development in response to stress, through mechanisms like ROS/RNS scavenging or persulfidation, ultimately adjusting stomatal density to reconcile growth and defense. Future work should prioritize mapping the precise spatiotemporal targets of H_2_S within the stomatal development network to fully elucidate its integrative signaling mechanism.

Our findings demonstrate that the SlLCD1-H_2_S preventing ROS over-accumulation via modulating the activities of antioxidant enzymes, albeit with distinct patterns depending on the specific enzyme (Fig. 6). H_2_S could orchestrate redox dynamics by differentially modulating antioxidant enzymes and ROS producers through protein persulfidation [98, 99]. Specifically, H_2_S-mediated persulfidation enhances APX activity [98], inhibits CAT activity [100, 101], activates RBOHD to induce the generation of signaling ROS [87, 102]. Although this persulfidation mechanistically links SlLCD1-H_2_S signaling to antioxidant activity, future studies are required to determine whether it directly regulates enzyme function under heat stress. ROS play a dual role in cell physiology, serving as a source of oxidative damage at high concentrations and as crucial signaling molecules at low levels. A well-established example of ROS signaling function is regulating stomatal movement [34, 36], which is finely tuned by redox homeostasis through key mechanisms such as the H_2_O_2_-brassinosteroid module for opening, ABA signaling for closure, and miR408-mediated integration of growth with drought resistance, etc. [34–36, 103]. Beyond stomatal regulation, ROS signaling (particularly chloroplast H_2_O_2_) acts as a central hub in the heat-stress response by directly activating the HSF-chaperone network [104]. Evidence includes H_2_O_2_-mediated induction of heat-responsive genes across species and transcriptional activation of specific small HSPs and HSFs [17, 105]. Based on these paradigms and our results, a compelling question arises: does the fine-tuning of ROS homeostasis by SlLCD1-H_2_S extend to the modulation of ROS signaling? We therefore propose that future research should focus on elucidating whether, and how, SlLCD1-H_2_S-mediated regulation calibrates the amplitude of ROS signals, and to what extent this calibration contributes to H_2_S-induced stomatal closure during heat stress. Additionally, it is critical to investigate whether SlLCD1-H_2_S enhances heat tolerance by modulating the HSP-HSF chaperone network, potentially through regulating ROS signaling. We believe that addressing these questions will provide fundamental insights for understanding of how H_2_S signaling coordinates stress responses and will significantly advance this field.

**Figure 9:**
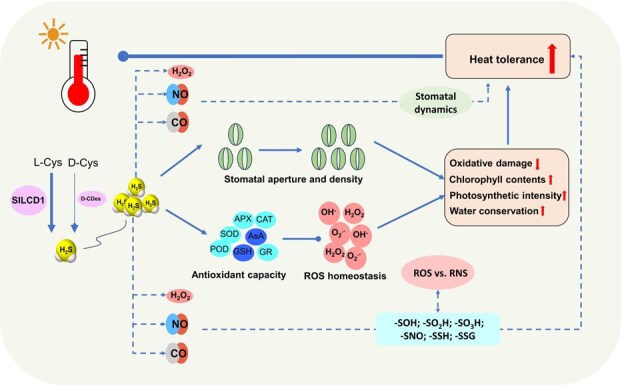
Model for SlLCD1-H_2_S module enhancing heat tolerance in tomato by enhancing antioxidant capacity and modulating stomatal morphology. The increase in SlLCD1-derived endogenous H_2_S activates a signaling pathway that functions as a central regulator of heat tolerance. It confers protection by a dual mechanism, enhancing the antioxidant capacity to prevent ROS overaccumulation, and fine-tuning stomatal morphology (aperture and density) to optimize the trade-off among transpirational cooling, water conservation, and photosynthetic efficiency. Moreover, H_2_S is proposed to function within a broader gasotransmitter network, where crosstalk with CO and NO modulates its signaling output during heat adaptation, and these hypothetical interactions are denoted by dashed lines.

Collectively, we propose a signaling model wherein the SlLCD1-H_2_S module enhances heat tolerance in tomato by maintaining redox homeostasis and coordinately regulating stomatal development and movement (Fig. 9). Upon exposure to extreme heat stress (44°C), the endogenous H_2_S generated by SlLCD1 is elevated, thereby activating H_2_S signaling. This signaling, on one hand, potentiates the antioxidant system to prevent the excessive accumulation of ROS and mitigate oxidative damage. On the other hand, it fine-tunes stomatal aperture and density to achieve an optimal balance among transpirational cooling, water conservation, and photosynthetic efficiency. Future studies need to investigate whether H_2_S-mediated modulation of redox balance integrates with ROS signaling to coordinate stomatal development and movement under heat stress. An integrative understanding requires analyzing H_2_S within the broader gasotransmitter network, especially its crosstalk with CO and NO, a feature that is also prevalent in heat stress responses. Specifically, H_2_S acts downstream of other gasotransmitters to induce thermotolerance: downstream of and synergistically with CO in tobacco cells, and downstream of NO in maize seedlings [61, 62]. Additionally, both CO and NO are known to regulate stomatal development and movement, often via ROS-mediated mechanisms [106–112]. Collectively, a plausible model is that H₂S enhances tomato heat tolerance via synergistic interactions with NO and CO, involving stomatal regulation and connections to ROS homeostasis or redox signaling (Fig. 9). Such crosstalk may occur via competitive or cooperative redox modifications of shared cysteine residues on stomatal regulatory or ROS scavenging proteins, including S-nitrosylation (by NO), persulfidation (by H_2_S), S-sulfenylation (by H_2_O_2_), and glutathionylation (by GSH) [102, 113–115]. Moving forward, studies should prioritize mapping the specific convergence points of these gasotransmitter pathways, particularly overlapping regulatory nodes dually modified by S-nitrosylation and persulfidation, to delineate a more integrated, multi-gas signaling network that orchestrates plant adaptation to dynamic environments.

### Limitations and future perspectives

Although this work advances our understanding of the genetic and mechanistic basis of the SlLCD1-H_2_S module in tomato heat adaptation, several limitations should be acknowledged when interpreting the findings. **(1) Constraints on cultivar scope and organ-specific focus.** Firstly, although Alisa Craig serves as a well-established model cultivar that minimizes genetic variability, exclusive reliance on this single cultivar limits the generalizability of the results. Therefore, validation of key findings across multiple commercial cultivars with distinct agronomic traits is essential to assess the broader applicability of the proposed mechanisms. Secondly, despite providing valuable mechanistic information, the exclusive focus on leaf responses limits direct horticultural translatability. Consequently, a key future objective is to systematically evaluate fruit-related parameters, such as yield, quality, and related molecular profiles, under heat stress conditions, using either the genetic materials or H_2_S application methods developed in this study. This approach will facilitate the linkage of mechanism to agronomic application. Moreover, establishing a systemic understanding of plant heat adaptation requires integrated multi-organ studies (e.g., roots for stress perception, vascular tissues for signaling, and flowers for reproductive development). Profiling transcriptomic, metabolic, and physiological changes across these organs under consistent experimental conditions is essential to elucidate the coordination of whole-plant heat stress responses. A rapid and essential validation step involves evaluating the heat-tolerance-promoting effects of exogenous H_2_S at two levels, across diverse genetic backgrounds and across multiple organs (e.g., fruit, roots, leaves), using integrated physiological assays. This naturally leads to another key limitation: **(2) Efficacy and safety of NaHS in field-based horticultural applications.** Although NaHS at physiological signaling concentrations is low and non-toxic, large-scale field application requires optimized formulations and thorough risk assessment. This is particularly critical given that environmental factors, such as soil properties, microbial activity, and light regimes, dynamically influence the bioavailability and bioactivity of H_2_S. Therefore, future research efforts should prioritize developing field-adaptable strategies, including slow-release H_2_S donors or genetic engineering approaches targeting key components of the pathway (e.g., *SlLCD1*). **(3) Limited field relevance of experimental heat stress conditions.** This study is primarily mechanistic, focusing on rapid heat-shock responses under controlled conditions to capture precise initial signaling and physiological events. While this experimental design provides key mechanistic insight, it does not directly replicate the prolonged or recurring heat stress typical of field environments. Nonetheless, the early-response pathways identified here, including rapid H_2_S signaling, ROS homeostasis rebalancing, stomatal adjustment, photosynthetic modulation, are likely crucial in initiating sustained acclimation. These pathways may prime transcriptional and metabolic programs for thermotolerance, fine-tune resource allocation and hormonal networks, and potentially establish systemic stress memory. Future studies should therefore integrate these early mechanisms into long-term, multi-stress field experiments to comprehensively evaluate and improve crop climate resilience.

Despite these limitations, this study establishes a foundation for future research and offers dual practical potential: serving as a tool for molecular breeding of heat-resilient crops, and providing a sustainable agronomic intervention that enhances water-use efficiency and reduces reliance on conventional protectants, aligning with the climate-smart agricultural objectives. To guide subsequent research, three priority directions are proposed: (1) Dissecting the specific contributions of H_2_S-synthesizing enzymes to clarify the endogenous sources and regulatory mechanisms of H_2_S signaling, followed by validation of their functional roles through the generation and phenotypic characterization of stable gene-edited mutants and complementary genetic lines. Although our H_2_S-based complementation experiments strongly support the ‘SlLCD1-H_2_S-heat tolerance’ model, definitive proof of gene function requires genetic complementation. Thus, the crucial subsequent step is to reintroduce SlLCD1 into the mutant background to obtain conclusive evidence. Consequently, producing and validating these genetic resources remains a central and essential aim for subsequent research. (2) Mapping the spatiotemporal dynamics of H_2_S signaling under heat stress conditions of varying intensities (severe vs. moderate) and investigating its potential role in heat stress memory. This should be coupled with integrated multi-omics analyses to predict key regulatory nodes and resolve functional differences across distinct stress scenarios. (3) Conducting long-term field trials under realistic, multi-stress conditions, an essential translational step to validate the agronomic relevance and resilience potential of manipulating the H_2_S pathway. Beyond these core directions, future research should also examine whether H_2_S signaling confers cross-tolerance to combined stresses (e.g., heat-drought or heat-pathogen interactions), thereby clarifying its role as a systemic integrator in environmental adaptation. Additionally, leveraging natural genetic variation in H_2_S-related genes across diverse crop germplasms could accelerate breeding of climate-resilient varieties. To deepen the mechanistic understanding, a more comprehensive experimental design, involving expanded sampling and time-resolved multi-parameter profiling, will enable rigorous correlation and path analysis. This, in turn, will quantify the relationships among H_2_S flux, ROS homeostasis, antioxidant responses, and photosynthetic performance under stress conditions.

## Materials and methods

### Plant material and growth condition

All experiments were performed using the tomato cultivar Ailsa Craig (AC). The *SlLCD1* mutant lines (*cr-sllcd1–6* and *cr-sllcd1–9*) were generated within the AC background using the CRISPR/Cas9 system, kindly provided by Yanxi Pei (Shanxi University) and Jixian Zhai (Southern University of Science and Technology). All subsequent phenotypic and mechanistic analyses were conducted using the homozygous and Cas9-free T3 *cr-sllcd1* mutants. For germination, seeds were first soaked in water at a temperature of 23°C for 2 h, followed by a 15-min immersion in 55°C warm water, and then soaked for an additional 5–6 h at 23°C before germination. Once the seeds had germinated and radicles emerged, they were transplanted into nursery pots filled with a 1:1 mixture of autoclaved peat and vermiculite. Seedlings were grown under a light intensity of 120 μmol m^−2^ s^−1^, 60% relative humidity, and a 16 h/8 h light/dark cycle. For the heat stress treatment, uniformly grown seedlings at the same growth/development stage (the six-true-leaf stage) were transferred from the control conditions (23°C) to an environment set at 44°C, while all other parameters remained identical. Prior to heat stress, a subset of plants (including *cr-sllcd1* mutants) underwent a pre-treatment with exogenous H_2_S via fumigation. The plants were sprayed uniformly with NaHS solution as an H_2_S donor, which releases H_2_S upon dissolution, and then immediately enclosed in transparent chambers to achieve and maintain a stable spatial H_2_S concentration of either 50 or 100 μM. All chambers were kept under standard growth conditions. Fumigation was performed nightly for 10 hours (10:00 p.m. to 8:00 a.m.) over three consecutive nights. Control groups were treated identically, except that an equivalent volume of water was used instead of the NaHS solution.

### Measurement of H_2_S content and enzymatic activity of CDes

Endogenous H_2_S levels and the enzymatic activity of CDes, including L-CDes and D-CDes, were measured according to our published methods [74, 75, 116]. Specifically, H_2_S content was quantified by the methylene blue method, which exploits the high reducibility of the predominant aqueous form of H_2_S, hydrosulfide (HS^−^), at physiological pH (pKa ≈ 7.0). CDes, including all L-Cys/D-Cys desulfhydrases or enzymes exhibiting such activities, catalyze the degradation of L/D-Cys to release H_2_S. Therefore, the enzymatic activity of L-CDes/D-CDes is defined as the amount of H_2_S produced per milligram of protein per minute when L-Cys or D-Cys is supplied as the substrate.

### Determination of the leaf RWC

The leaf RWC was determined according to a previously published method [82]. Briefly, fresh tomato leaves were sampled and their fresh weight (FW) was immediately recorded. The leaves were then floated on distilled water under low light for 4 h to achieve full turgidity, after which the saturated weight (SW) was measured. Subsequently, the leaves were oven-dried at 65°C for 24 h, with their weight monitored continuously until it stabilized (i.e., no change over three consecutive measurements), and the constant weight was recorded as the dry weight (DW). The RWC was calculated as follows: RWC (%) = [(FW – DW) / (SW – DW)] × 100%.

### Measurement of photosynthetic pigment concentration and photosynthetic characteristics

Leaf tissue (0.1 g) was homogenized in 1 mL of 95% ethanol, followed by centrifugation to collect the supernatant. An additional 3 mL of 95% ethanol was added to the supernatant, and the mixture was thoroughly vortexed, sealed, and extracted in darkness at room temperature for 24–36 hours until the tissue became completely bleached. Absorbance was measured at 665 nm and 649 nm using a UV spectrophotometer [82]. The concentrations of chlorophyll a, chlorophyll b, and total chlorophyll (in mg L^−1^) were calculated from the absorbance values according to Arnon’s equations, as follows: Chl. a (mg L^−1^) = 13.95 × A_665_ – 6.88 × A_649_; Chl. b (mg L^−1^) = 24.96 × A_649_ – 7.32 × A_665_; Total Chl. (mg L^−1^) = 6.63 × A_665_ + 18.08 × A_649_. The pigment content was then normalized to the fresh weight and expressed as mg per gram of fresh weight (mg g^−1^ FW).

The photosynthetic characteristics, such as Pn, Ci, Gs, and Tr were monitored with a LI-6800 Portable Photosynthesis System (LI-COR, Nebraska, USA). The maximum quantum efficiency of PSII photochemistry (Fv/Fm) was analyzed with PAM-2500 chlorophyll fluorometer (WALZ, Germany). For each treatment, six independent plants were measured by randomly sampling three leaves from the 4^th^ to 6^th^ leaf position per plant. One measurement per leaf was recorded, yielding 18 biological replicates per treatment. Data are presented as mean ± SE.

### Determination of the level of ROS and the degree of oxidative damage

The histochemical detection of H_2_O_2_ was performed using 3,3′-diaminobenzidine (DAB), which produces a brown-red precipitate upon reaction with H_2_O_2_, following a published protocol [117]. Meanwhile, the levels of superoxide anion radical (O_2_^−^) and H_2_O_2_ in leaves were quantified using their respective commercial assay kits.

MDA, a terminal product of lipid peroxidation, was measured as an indicator of oxidative damage using the thiobarbituric acid (TBA) method [118]. Briefly, 0.15 g of tomato leaf tissue was homogenized in 1.5 mL of 5% trichloroacetic acid (TCA) on ice. The homogenate was centrifuged at 5000 rpm for 5 min at 4°C. Subsequently, 1 mL of the supernatant was reacted with an equal volume of 0.67% TBA solution. The mixture was incubated in a boiling water bath for 30 min, cooled, and centrifuged. The absorbance of the resulting supernatant was measured at 450, 532, and 600 nm. The MDA content was calculated using the following formula, MDA (μmol g^−1^ FW) = [6.45 × (A_532_ − A_600_) − 0.56 × A_450_] × V/(0.15 g FW). REL, an indicator of oxidative damage to cell membranes, was determined as described previously. Uniform leaf discs were rinsed with distilled water, blotted dry, and cut into small pieces (midvein excluded). A 0.1 g sample was immersed in 10 mL of deionized water and incubated at room temperature for 6 h. The initial conductivity (R_1_) was recorded. After heating the samples in a boiling water bath for 40 min and cooling to room temperature, the final conductivity (R_2_) was measured. REL was calculated using the formula: REL (%) = (R_1_/R_2_) × 100%.

### Assay of activities of antioxidant enzymes and contents of antioxidants

Approximately 0.1 g of plant tissue was weighed and thoroughly homogenized in 1 mL of 0.05 mol/L PBS (pH 7.0) on ice. The homogenate was then centrifuged at 12000 rpm for 5 min at 4°C. The resulting supernatant was collected and used as the crude enzyme extract for the determination of the activities of APX, POD, CAT, SOD, and GR using specific commercial assay kits. The contents of reduced GSH and AsA, were also measured with corresponding commercial assay kits.

### Measurement of stomatal density and stomatal aperture

Stomatal density and aperture were analyzed on the abaxial epidermis of leaves collected from the same nodal position. The epidermis was rapidly sampled using transparent adhesive tape and fixed with absolute ethanol. Stomatal density was assessed under a light microscope at 100× total magnification (10× ocular and 10× objective), expressed as the number of stomata per mm^2^. For aperture measurement, images were captured at 400× magnification (10× ocular and 40× objective). Each treatment included three biological replicates. For each replicate, three randomly selected fields of view were analyzed, in which the total stomatal count was recorded and the aperture size of 20 stomata was measured using ImageJ software.

For the isolated epidermal strip assay, lower epidermal strips of tomato leaves were first incubated for 2 hours in a buffer solution (10 mM MES, 50 mM KCl, pH 6.15) under a plant-culture lamp [119, 120]; these served as the CK samples. Subsequently, the strips were exposed to heat stress for an additional 2 h under different treatment conditions, either in the absence or presence of 1 mM H_2_O_2_ or 100 U/L CAT [121, 122]. After treatment, strips were immediately fixed in absolute ethanol for 20 min to preserve stomatal apertures, and then imaged. Each treatment was performed in three biological replicates. Per replicate, stomatal apertures were measured from 20 stomata located in three random fields of view per leaf using ImageJ software.

### Total RNA extraction and RT-qPCR

Total RNA was extracted from samples using the FastPure® Universal Plant Total RNA Isolation Kit. Subsequently, first-strand cDNA was synthesized from the extracted RNA using the PrimerScript II First-Strand cDNA Synthesis Kit (TaKaRa). The resulting cDNA was diluted 10-fold and subjected to RT-qPCR analysis using 2× NovoStart® SYBR qPCR SuperMix Plus on a qTOWER3/G Real-Time PCR System. Gene-specific primers (Table S2) were validated by standard-curve analysis (using serial 5-fold dilutions from 5^−1^ to 5^−5^) and melt-curve assessment. All assays demonstrated robust performance, with amplification efficiencies between 100% and 110% and all standard curves yielding a coefficient of determination (R^2^) > 0.99. Relative expression levels were calculated using the 2^–ΔΔCt^ method, with data from three independent biological replicates presented as mean ± SE.

### Statistical analysis

All experiments were conducted with three biological replicates. Data were presented as mean ± standard error (SE). Statistical significance among groups was determined by one-way analysis of variance (ANOVA) followed by Tukey’s multiple comparison test using SPSS software (version 25.0). Differences were considered statistically significant at *p* < 0.05.

## Supplementary Material

Web_Material_uhag090

## Data Availability

All data generated or analyzed during this study are included in this present article.

## References

[1] WORLD METEOROLOGICAL ORGANIZATION (WMO) . Global temperature is likely to exceed 1.5°C above pre-industrial level temporarily in next 5 years. 2024. https://wmo.int/publication-series/state-of-climate-arab-region-2024.

[2] Tollefson J . IPCC climate report: earth is warmer than it's been in 125,000 years. Nature. 2021;596:171–2 34373637 10.1038/d41586-021-02179-1

[3] IPCC *. Sixth Assessment Report of the Intergovernmental Panel on Climate Change Working Group II Observed and Projected Impact Assessment Database v1.0. NASA Socioeconomic Data and Applications Center (SEDAC), IPCC DDC*. 2023.

[4] Hultgren A, Carleton T, Delgado M. et al. Impacts of climate change on global agriculture accounting for adaptation. Nature. 2025;642:644–52 40533541 10.1038/s41586-025-09085-w PMC12176627

[5] Dorais M, Papadopoulos AP, Gosselin A. Greenhouse tomato fruit quality. Hortic Rev. 2001;26:239–319

[6] Hu T, Xuesong Z, Khanal S. et al. Climate change impacts on crop yields: a review of empirical findings, statistical crop models, and machine learning methods. Environ Model Softw. 2024;179:106119

[7] Hendrix S, Dard A, Meyer AJ. et al. Redox-mediated responses to high temperature in plants. J Exp Bot. 2023;74:2489–507 36794477 10.1093/jxb/erad053

[8] Kan Y, Mu XR, Gao J. et al. The molecular basis of heat stress responses in plants. Mol Plant. 2023;16:1612–34 37740489 10.1016/j.molp.2023.09.013

[9] Liu Y, Sun Y, Lv P. et al. Morphological, physiological, and molecular mechanisms underlying heat stress memory in plants. Vegetable *Res*. 2025;5:e035

[10] Huang X, Xiao N, Xie Y. et al. ROS burst prolongs transcriptional condensation to slow shoot apical meristem maturation and achieve heat-stress resilience in tomato. Dev Cell. 2025;60:2032–2045.e3 40179886 10.1016/j.devcel.2025.03.007

[11] Wang P, Liu WC, Han C. et al. Reactive oxygen species: multidimensional regulators of plant adaptation to abiotic stress and development. J Integr Plant Biol. 2024;66:330–67 38116735 10.1111/jipb.13601

[12] Wang Y, Sun S, Feng X. et al. Two lncRNAs of Chinese cabbage confer Arabidopsis with heat and drought tolerance. Vegetable *Res*. 2024;4:e029

[13] Dickinson PJ, Kumar M, Martinho C. et al. Chloroplast signaling gates thermotolerance in Arabidopsis. Cell Rep. 2018;22:1657–65 29444421 10.1016/j.celrep.2018.01.054 PMC5847188

[14] Mittler R, Vanderauwera S, Suzuki N. et al. ROS signaling: the new wave? Trends Plant Sci. 2011;16:300–9 21482172 10.1016/j.tplants.2011.03.007

[15] Miller G, Mittler R. Could heat shock transcription factors function as hydrogen peroxide sensors in plants? Ann Bot. 2006;98:279–88 16740587 10.1093/aob/mcl107 PMC2803459

[16] Suzuki N, Koussevitzky S, Mittler R. et al. ROS and redox signalling in the response of plants to abiotic stress. Plant Cell Environ. 2012;35:259–70 21486305 10.1111/j.1365-3040.2011.02336.x

[17] Banzet N, Richaud C, Deveaux Y. et al. Accumulation of small heat shock proteins, including mitochondrial HSP22, induced by oxidative stress and adaptive response in tomato cells. Plant J. 1998;13:519–27 9680997 10.1046/j.1365-313x.1998.00056.x

[18] Kotak S, Larkindale J, Lee U. et al. Complexity of the heat stress response in plants. Curr Opin Plant Biol. 2007;10:310–6 17482504 10.1016/j.pbi.2007.04.011

[19] Kovtun Y, Chiu WL, Tena G. et al. Functional analysis of oxidative stress-activated mitogen-activated protein kinase cascade in plants. Proc Natl Acad Sci USA. 2000;97:2940–5 10717008 10.1073/pnas.97.6.2940 PMC16034

[20] Saidi Y, Finka A, Goloubinoff P. Heat perception and signalling in plants: a tortuous path to thermotolerance. New Phytol. 2011;190:556–65 21138439 10.1111/j.1469-8137.2010.03571.x

[21] Volkov RA, Panchuk II, Mullineaux PM. et al. Heat stress-induced H_2_O_2_ is required for effective expression of heat shock genes in Arabidopsis. Plant Mol Biol. 2006;61:733–46 16897488 10.1007/s11103-006-0045-4

[22] Peláez Vico MN, Zandalinas SI, Devireddy AR. et al. Systemic stomatal responses in plants: coordinating development, stress, and pathogen defense under a changing climate. Plant Cell Environ. 2024;47:1171–84 38164061 10.1111/pce.14797

[23] Rizhsky L, Liang H, Shuman J. et al. When defense pathways collide. The response of Arabidopsis to a combination of drought and heat stress. Plant Physiol. 2004;134:1683–96 15047901 10.1104/pp.103.033431 PMC419842

[24] Sato H, Mizoi J, Shinozaki K. et al. Complex plant responses to drought and heat stress under climate change. Plant J. 2024;117:1873–92 38168757 10.1111/tpj.16612

[25] Caine RS, Yin X, Sloan J. et al. Rice with reduced stomatal density conserves water and has improved drought tolerance under future climate conditions. New Phytol. 2018;221:371–84 30043395 10.1111/nph.15344 PMC6492113

[26] Jiang T, Feng Y, Zhao M. et al. Regulation mechanism of melatonin on photosynthesis of cucumber under high temperature stress. Vegetable *Res*. 2024;4:e037

[27] Drake JE, Tjoelker MG, Varhammar A. et al. Trees tolerate an extreme heatwave via sustained transpirational cooling and increased leaf thermal tolerance. Glob Chang Biol. 2018;24:2390–402 29316093 10.1111/gcb.14037

[28] Ilan N, Moran N, Schwartz A. The role of potassium channels in the temperature control of stomatal aperture. Plant Physiol. 1995;108:1161–70 12228534 10.1104/pp.108.3.1161 PMC157469

[29] Kostaki KI, Coupel-Ledru A, Bonnell VC. et al. Guard cells integrate light and temperature signals to control stomatal aperture. Plant Physiol. 2020;182:1404–19 31949030 10.1104/pp.19.01528 PMC7054865

[30] Liu C, Sack L, Li Y. et al. Relationships of stomatal morphology to the environment across plant communities. Nat Commun. 2023;14:6629 37857672 10.1038/s41467-023-42136-2 PMC10587080

[31] Sinha R, Shostak B, Induri SP. et al. Differential transpiration between pods and leaves during stress combination in soybean. Plant Physiol. 2023;192:753–66 36810691 10.1093/plphys/kiad114 PMC10231362

[32] Sinha R, Zandalinas SI, Fichman Y. et al. Differential regulation of flower transpiration during abiotic stress in annual plants. New Phytol. 2022;235:611–29 35441705 10.1111/nph.18162 PMC9323482

[33] Teskey R, Wertin T, Bauweraerts I. et al. Responses of tree species to heat waves and extreme heat events. Plant Cell Environ. 2015;38:1699–712 25065257 10.1111/pce.12417

[34] da Silva WA, Goncalves JP, Martins AO. et al. Open or closed: ROS-mediated regulation of stomatal movements. Trends Plant Sci. 2025;30:1292–4 40803978 10.1016/j.tplants.2025.08.003

[35] Shi W, Liu Y, Zhao N. et al. Hydrogen peroxide is required for light-induced stomatal opening across different plant species. Nat Commun. 2024;15:5081 38876991 10.1038/s41467-024-49377-9 PMC11178795

[36] Sierla M, Waszczak C, Vahisalu T. et al. Reactive oxygen species in the regulation of stomatal movements. Plant Physiol. 2016;171:1569–80 27208297 10.1104/pp.16.00328 PMC4936562

[37] Chen J, Zhou H, Xie Y. SnRK2.6 phosphorylation/persulfidation: where ABA and H_2_S signaling meet. Trends Plant Sci. 2021;26:1207–9 34493443 10.1016/j.tplants.2021.08.005

[38] Liu H, Xue S. Interplay between hydrogen sulfide and other signaling molecules in the regulation of guard cell signaling and abiotic/biotic stress response. Plant Commun. 2021;2:100179 34027393 10.1016/j.xplc.2021.100179 PMC8132131

[39] Zhang J, Zhou M, Ge Z. et al. Abscisic acid-triggered guard cell L-cysteine desulfhydrase function and in situ hydrogen sulfide production contributes to heme oxygenase-modulated stomatal closure. Plant Cell Environ. 2020;43:624–36 31734942 10.1111/pce.13685

[40] Yin Y, Hayashi Y, Sirajam M. et al. ABA receptor isoforms differently regulate stomatal movements and generation of reactive oxygen species in ABA signaling in Arabidopsis guard cells. Plant Cell Physiol. 2025;66:1811–22 40874627 10.1093/pcp/pcaf102 PMC12739107

[41] Wang R . Gasotransmitters: growing pains and joys. Trends Biochem Sci. 2014;39:227–32 24767680 10.1016/j.tibs.2014.03.003

[42] Hao X, Li W, Cao H. et al. H_2_S promotes flowering in Chinese cabbage by persulfidation of the splicing factor BraATO2. Hortic Res. 2025;12:uhaf190 41127271 10.1093/hr/uhaf190 PMC12539866

[43] Hu H, Qin M, Zhang J. et al. H_2_S-mediated protein S-sulfhydration modulates infectivity and autophagy in the rice blast fungus. Nat Commun. 2025;16:6222 40617858 10.1038/s41467-025-61582-8 PMC12228824

[44] Liu H, Liang X, Liu R. et al. Hydrogen sulfide inhibits Arabidopsis inward potassium channels via protein persulfidation. J Integr Plant Biol. 2025;67:1217–9 39888262 10.1111/jipb.13851

[45] Liu T, Chen H, Luo S. et al. Hydrogen sulphide alleviates root growth inhibition induced by phosphate starvation. Plant Cell Environ. 2024;47:5265–79 39175420 10.1111/pce.15110

[46] Ma T, Xu S, Wang Y. et al. Exogenous hydrogen sulphide promotes plant flowering through the Arabidopsis splicing factor AtU2AF65a. Plant Cell Environ. 2024;47:1782–96 38315745 10.1111/pce.14849

[47] Zhang J, Li H, Xie Y. A paradigm shift in stomatal regulation: H_2_S as a persulfidation-driven modulator of potassium channels. J Integr Plant Biol. 2025;67:1433–4 40062733 10.1111/jipb.13886

[48] Zhang J, Zhou M, Zhou H. et al. Hydrogen sulfide, a signaling molecule in plant stress responses. J Integr Plant Biol. 2021;63:146–60 33058490 10.1111/jipb.13022

[49] Zhou M, Xie Y. Mitochondrial H_2_S production regulates stomatal immunity. Plant Cell Environ. 2025;48:1215–6 39420656 10.1111/pce.15234

[50] Hu KD, Zhang XY, Yao GF. et al. A nuclear-localized cysteine desulfhydrase plays a role in fruit ripening in tomato. Hortic Res. 2020;7:211 33328464 10.1038/s41438-020-00439-1 PMC7736880

[51] Wang R . Two's company, three's a crowd: can H_2_S be the third endogenous gaseous transmitter? FASEB J. 2002;16:1792–8 12409322 10.1096/fj.02-0211hyp

[52] Liu H, Wang J, Liu J. et al. Hydrogen sulfide (H_2_S) signaling in plant development and stress responses. aBIOTECH. 2021;2:32–63 34377579 10.1007/s42994-021-00035-4 PMC7917380

[53] Alvarez C, Calo L, Romero LC. et al. An O-acetylserine(thiol)lyase homolog with L-cysteine desulfhydrase activity regulates cysteine homeostasis in Arabidopsis. Plant Physiol. 2010;152:656–69 19955263 10.1104/pp.109.147975 PMC2815857

[54] Romero LC, Aroca MA, Laureano-Marin AM. et al. Cysteine and cysteine-related signaling pathways in *Arabidopsis thaliana*. Mol Plant. 2014;7:264–76 24285094 10.1093/mp/sst168

[55] Christou A, Filippou P, Manganaris GA. et al. Sodium hydrosulfide induces systemic thermotolerance to strawberry plants through transcriptional regulation of heat shock proteins and aquaporin. BMC Plant Biol. 2014;14:42 24499299 10.1186/1471-2229-14-42 PMC3933230

[56] Min Y, Bao-Ping Q, Xue-Li MA. et al. Foliar application of sodium hydrosulfide (NaHS), a hydrogen sulfide (H_2_S) donor, can protect seedlings against heat stress in wheat (*Triticum aestivum* L.). J Integr Agric. 2016;15:2745–58

[57] Ye XY, Qiu XM, Sun YY. et al. Interplay between hydrogen sulfide and methylglyoxal initiates thermotolerance in maize seedlings by modulating reactive oxidative species and osmolyte metabolism. Protoplasma. 2020;257:1415–32 32474849 10.1007/s00709-020-01516-x

[58] Li ZG, Ding XJ, Du PF. Hydrogen sulfide donor sodium hydrosulfide-improved heat tolerance in maize and involvement of proline. J Plant Physiol. 2013;170:741–7 23523123 10.1016/j.jplph.2012.12.018

[59] Li ZG, Gong M, Xie H. et al. Hydrogen sulfide donor sodium hydrosulfide-induced heat tolerance in tobacco (*Nicotiana tabacum* L) suspension cultured cells and involvement of Ca^2+^ and calmodulin. Plant Sci. 2012;185-186:185–9 22325880 10.1016/j.plantsci.2011.10.006

[60] Feng L, Wei L, Liu Y. et al. Carbon monoxide/heme oxygenase system in plant: roles in abiotic stress response and crosstalk with other signals molecules. Nitric Oxide. 2023;138-139:51–63 37364740 10.1016/j.niox.2023.06.005

[61] Li ZG, Gu SP. Hydrogen sulfide as a signal molecule in hematin-induced heat tolerance of tobacco cell suspension. Biol Plant. 2016;60:595–600

[62] Li ZG, Yang SZ, Long WB. et al. Hydrogen sulphide may be a novel downstream signal molecule in nitric oxide-induced heat tolerance of maize (*Zea mays* L.) seedlings. Plant Cell Environ. 2013;36:1564–72 23489239 10.1111/pce.12092

[63] Wang R . Physiological implications of hydrogen sulfide: a whiff exploration that blossomed. Physiol Rev. 2012;92:791–896 22535897 10.1152/physrev.00017.2011

[64] Liu H, Ding Y, Zhou Y. et al. CRISPR-P 2.0: an improved CRISPR-Cas9 tool for Genome editing in plants. Mol Plant. 2017;10:530–2 28089950 10.1016/j.molp.2017.01.003

[65] Fang H, Zhang X, Chen W. et al. Dynamic changes of endogenous H_2_S generation during responding to developmental and environmental signals in *Solanum lycopersicum* L. Sci Hortic. 2024;335:113346

[66] Liu D, Lu J, Li H. et al. Characterization of the O-acetylserine(thiol)lyase gene family in *Solanum lycopersicum* L. Plant Mol Biol. 2019;99:123–34 30535734 10.1007/s11103-018-0807-9

[67] Wilson LG, Bressan RA, Filner P. Light-dependent emission of hydrogen sulfide from plants. Plant Physiol. 1978;61:184–9 16660257 10.1104/pp.61.2.184 PMC1091829

[68] Harrington HM, Smith IK. Cysteine metabolism in cultured tobacco cells. Plant Physiol. 1980;65:151–5 16661132 10.1104/pp.65.1.151 PMC440285

[69] Nagasawa T, Ishii T, Kumagai H. et al. D-cysteine desulfhydrase of *Escherichia coli. Purification and characterization*. Eur J Biochem. 1985;153:541–51 3908101 10.1111/j.1432-1033.1985.tb09335.x

[70] Rennenberg H, Filner P. Developmental changes in the potential for H_2_S emission in cucurbit plants. Plant Physiol. 1983;71:269–75 16662816 10.1104/pp.71.2.269 PMC1066023

[71] Riemenschneider A, Wegele R, Schmidt A. et al. Isolation and characterization of a D-cysteine desulfhydrase protein from *Arabidopsis thaliana*. FEBS J. 2005;272:1291–304 15720402 10.1111/j.1742-4658.2005.04567.x

[72] Cao H, Liang Y, Zhang L. et al. AtPRMT5-mediated AtLCD methylation improves Cd^2+^ tolerance via increased H_2_S production in Arabidopsis. Plant Physiol. 2022;190:2637–50 35972421 10.1093/plphys/kiac376 PMC9706440

[73] Fang H, Liu R, Yu Z. et al. Gasotransmitter H_2_S accelerates seed germination via activating AOX mediated cyanide-resistant respiration pathway. Plant Physiol Biochem. 2022;190:193–202 36126464 10.1016/j.plaphy.2022.09.003

[74] Fang H, Liu Z, Long Y. et al. The Ca^2+^/calmodulin2-binding transcription factor TGA3 elevates *LCD* expression and H_2_S production to bolster Cr^6+^ tolerance in Arabidopsis. Plant J. 2017;91:1038–50 28670772 10.1111/tpj.13627

[75] Jin Z, Wang Z, Ma Q. et al. Hydrogen sulfide mediates ion fluxes inducing stomatal closure in response to drought stress in *Arabidopsis thaliana*. Plant Soil. 2017;419:141–52

[76] Jin Z, Xue S, Luo Y. et al. Hydrogen sulfide interacting with abscisic acid in stomatal regulation responses to drought stress in Arabidopsis. Plant Physiol Biochem. 2013;62:41–6 23178483 10.1016/j.plaphy.2012.10.017

[77] Zhou M, Zhang K, Xie Y. Revealing how plants utilize H_2_S to relay drought stress signals. Trends Plant Sci. 2025;30:13–6 39332914 10.1016/j.tplants.2024.09.002

[78] Shen J, Su Y, Zhou C. et al. A putative rice L-cysteine desulfhydrase encodes a true L-cysteine synthase that regulates plant cadmium tolerance. Plant Growth Regul. 2019;89:217–26

[79] Zhao YQ, Hu KD, Yao GF. et al. A D-cysteine desulfhydrase, SlDCD2, participates in tomato fruit ripening by modulating ROS homoeostasis and ethylene biosynthesis. Hortic Res. 2023;10:uhad14 10.1093/hr/uhad014 PMC10031741 36968183

[80] Birke H, Hildebrandt TM, Wirtz M. et al. Sulfide detoxification in plant mitochondria. Methods Enzymol. 2015;555:271–86 25747485 10.1016/bs.mie.2014.11.027

[81] Stanislav K, Parisa RK, Hideki T. Adaptive modifications in plant sulfur metabolism over evolutionary time. J Exp Bot. 2024;75:4697–711 38841807 10.1093/jxb/erae252 PMC11350084

[82] Fang H, Chen W, Xing K. et al. Hydrogen sulfide generated by L-cysteine desulfhydrase 1 enhances plant basal thermotolerance through the regulation of stomatal behavior and the promotion of photosynthetic efficiency. Plant Sci. 2025;361:112805 41067562 10.1016/j.plantsci.2025.112805

[83] Mishra SK, Tripp J, Winkelhaus S. et al. In the complex family of heat stress transcription factors, HsfA1 has a unique role as master regulator of thermotolerance in tomato. Genes Dev. 2002;16:1555–67 12080093 10.1101/gad.228802 PMC186353

[84] Rao S, Das JR, Mathur S. Exploring the master regulator heat stress transcription factor HSFA1a-mediated transcriptional cascade of HSFs in the heat stress response of tomato. J Plant Biochem Biotechnol. 2021;30:878–88

[85] Xie DL, Huang HM, Zhou CY. et al. HsfA1a confers pollen thermotolerance through upregulating antioxidant capacity, protein repair, and degradation in *Solanum lycopersicum* L. Hortic Res. 2022;9:uhac163 36204210 10.1093/hr/uhac163 PMC9531336

[86] Chen S, Jia H, Wang X. et al. Hydrogen sulfide positively regulates abscisic acid signaling through persulfidation of SnRK2.6 in guard cells. Mol Plant. 2020;13:732–44 31958520 10.1016/j.molp.2020.01.004

[87] Shen J, Zhang J, Zhou M. et al. Persulfidation-based modification of cysteine desulfhydrase and the NADPH oxidase RBOHD controls guard cell abscisic acid signaling. Plant Cell. 2020;32:1000–17 32024687 10.1105/tpc.19.00826 PMC7145499

[88] Wang Z, Mu Y, Zhang L. et al. Hydrogen sulfide mediated the melatonin induced stoma closure by regulating the K^+^ channel in *Arabidopsis thaliana*. Environ Exp Bot. 2022;205:105125

[89] Wang L, Wan R, Shi Y. et al. Hydrogen sulfide activates S-type anion channel via OST1 and Ca^2+^ modules. Mol Plant. 2016;9:489–91 26678664 10.1016/j.molp.2015.11.010

[90] Zhang J, Zhang L, Han X. et al. H_2_S modulates BrSDH1-1 alternative splicing to prompt stomatal closure in Chinese cabbage. Hortic Res. 2025;12:uhaf214 41189981 10.1093/hr/uhaf214 PMC12580989

[91] Zhang J, Zhang L, Zhang W. et al. H_2_S activates succinate dehydrogenase through persulfidation to induce stomatal closure in Arabidopsis. Plant Soil. 2025;508:939–54

[92] Jin Z . From odor to order: unveiling the crucial role of hydrogen sulfide in plant life. Hortic Res. 2025;13:uhaf273 41659372 10.1093/hr/uhaf273 PMC12881861

[93] Deng G, Zhou L, Wang Y. et al. Hydrogen sulfide acts downstream of jasmonic acid to inhibit stomatal development in Arabidopsis. Planta. 2020;251:42 31907619 10.1007/s00425-019-03334-9

[94] Jin Z, Garcia-Mata C, Pei Y. Hydrogen sulfide: a tiny molecule with a big role in stomatal regulation. J Plant Physiol. 2025;311:154539 40499394 10.1016/j.jplph.2025.154539

[95] Xue S, Corpas FJ, Modolo LV. et al. Small molecules and ions: minor yet vital in plants. J Plant Physiol. 2025;306:154451 39951840 10.1016/j.jplph.2025.154451

[96] Bo S, Li C, Zhang J. et al. Hydrogen sulfide promotes stomatal development through persulfidation of EPF peptides in Arabidopsis. Plant Physiol Biochem. 2025;227:110171 40609392 10.1016/j.plaphy.2025.110171

[97] Duan B, Ma Y, Jiang M. et al. Improvement of photosynthesis in rice (*Oryza sativa* L.) as a result of an increase in stomatal aperture and density by exogenous hydrogen sulfide treatment. Plant Growth Regul. 2015;75:33–44

[98] Aroca A, Serna A, Gotor C. et al. S-sulfhydration: a cysteine posttranslational modification in plant systems. Plant Physiol. 2015;168:334–42 25810097 10.1104/pp.15.00009 PMC4424021

[99] Chen T, Tian M, Han Y. Hydrogen sulfide: a multi-tasking signal molecule in the regulation of oxidative stress responses. J Exp Bot. 2020;71:2862–9 32076713 10.1093/jxb/eraa093

[100] Corpas FJ, Barroso JB, Gonzalez-Gordo S. et al. Hydrogen sulfide: a novel component in Arabidopsis peroxisomes which triggers catalase inhibition. J Integr Plant Biol. 2019;61:871–83 30652411 10.1111/jipb.12779

[101] Palma JM, Mateos RM, Lopez-Jaramillo J. et al. Plant catalases as NO and H_2_S targets. Redox Biol. 2020;34:101525 32505768 10.1016/j.redox.2020.101525 PMC7276441

[102] Zhang W, Liu W, Wang K. et al. Persulfidation of host NADPH oxidase RbohB by rhizobial 3-mercaptopyruvate sulfurtransferase maintains redox homeostasis and promotes symbiotic nodulation in soybean. Mol Plant. 2025;18:1843–63 40958419 10.1016/j.molp.2025.09.013

[103] Yang Y, Xu L, Hao C. et al. The microRNA408-plantacyanin module balances plant growth and drought resistance by regulating reactive oxygen species homeostasis in guard cells. Plant Cell. 2024;36:4338–55 38723161 10.1093/plcell/koae144 PMC11448907

[104] Mittler R, Zandalinas SI, Fichman Y. et al. Reactive oxygen species signalling in plant stress responses. Nat Rev Mol Cell Biol. 2022;23:663–79 35760900 10.1038/s41580-022-00499-2

[105] Crawford T, Lehotai N, Strand A. The role of retrograde signals during plant stress responses. J Exp Bot. 2018;69:2783–95 29281071 10.1093/jxb/erx481

[106] Desikan R, Cheung M, Bright J. et al. ABA, hydrogen peroxide and nitric oxide signalling in stomatal guard cells. J Exp Bot. 2004;55:205–12 14673026 10.1093/jxb/erh033

[107] Song X, She X, Zhang B. Carbon monoxide-induced stomatal closure in *Vicia faba* is dependent on nitric oxide synthesis. Physiol Plant. 2008;132:514–25 18334004 10.1111/j.1399-3054.2007.01026.x

[108] Sun LR, Yue CM, Hao FS. Update on roles of nitric oxide in regulating stomatal closure. Plant Signal Behav. 2019;14:e1649569 31370725 10.1080/15592324.2019.1649569 PMC6768244

[109] Wang P, Du Y, Hou Y. et al. Nitric oxide negatively regulates abscisic acid signaling in guard cells by S-nitrosylation of OST1. Proc Natl Acad Sci USA. 2015;112:613–8 25550508 10.1073/pnas.1423481112 PMC4299189

[110] Weng X, Zhu L, Yu S. et al. Carbon monoxide promotes stomatal initiation by regulating the expression of two *EPF* genes in Arabidopsis cotyledons. Front. Plant Sci. 2022;13:1029703 10.3389/fpls.2022.1029703 PMC9691970 36438138

[111] Xie Y, Mao Y, Duan X. et al. Arabidopsis HY1-modulated stomatal movement: an integrative hub is functionally associated with ABI4 in dehydration-induced ABA responsiveness. Plant Physiol. 2016;170:1699–713 26704641 10.1104/pp.15.01550 PMC4775125

[112] Cao Z, Huang B, Wang Q. et al. Involvement of carbon monoxide produced by heme oxygenase in ABA-induced stomatal closure in *Vicia faba* and its proposed signal transduction pathway. Chin Sci Bull. 2007;52:2365–73

[113] Corpas FJ, Gonzalez-Gordo S, Rodriguez-Ruiz M. et al. Thiol-based oxidative posttranslational modifications (OxiPTMs) of plant proteins. Plant Cell Physiol. 2022;63:889–900 35323963 10.1093/pcp/pcac036 PMC9282725

[114] Jimenez A, Marti MC, Sevilla F. Oxidative post-translational modifications of plant antioxidant systems under environmental stress. Physiol Plant. 2025;177:e70118 39968905 10.1111/ppl.70118 PMC11837463

[115] Mohammadbagherlou S, Samari E, Sagharyan M. et al. Hydrogen sulfide mechanism of action in plants; from interaction with regulatory molecules to persulfidation of proteins. Nitric Oxide. 2025;156:27–41 40024432 10.1016/j.niox.2025.02.001

[116] Fang H, Zang Y. An overview of analytical methods for detecting endogenous hydrogen sulfide (H_2_S) in plants. J Plant Physiol. 2024;302:154315 39053091 10.1016/j.jplph.2024.154315

[117] Kumar D, Yusuf MA, Singh P. et al. Histochemical detection of superoxide and H_2_O_2_ accumulation in *Brassica juncea* seedlings. Bio Protoc. 2014;4:1108

[118] Ganh OR, Estévez M, Morcuende D. Suitability of the TBA method for assessing lipid oxidation in a meat system with added phenolic-rich materials. Food Chem. 2011;126:772–8

[119] Zhang L, Li D, Yao Y. et al. H_2_O_2_, Ca^2+^, and K^+^ in subsidiary cells of maize leaves are involved in regulatory signaling of stomatal movement. Plant Physiol Biochem. 2020;152:243–51 32449683 10.1016/j.plaphy.2020.04.045

[120] Zhang W, Wang L, Zhang L. et al. H_2_S-mediated balance regulation of stomatal and non-stomatal factors responding to drought stress in Chinese cabbage. Hortic Res. 2023;10:uhac284 36938567 10.1093/hr/uhac284 PMC10018781

[121] Lv S, Zhang Y, Li C. et al. Strigolactone-triggered stomatal closure requires hydrogen peroxide synthesis and nitric oxide production in an abscisic acid-independent manner. New Phytol. 2018;217:290–304 28940201 10.1111/nph.14813

[122] Scuffi D, Pantaleno R, Schiel P. et al. Hydrogen sulfide modulates flagellin-induced stomatal immunity. Plant Physiol. 2025;199:kiaf561 41432548 10.1093/plphys/kiaf561

