# Supplementary material for: H2S generated by L-cysteine desulfhydrase (SlLCD1) enhances heat tolerance in tomato via antioxidant capacity and stomatal modulation
Source: Hortic Res. 2026 Mar 9;13(7):uhag090. doi: 10.1093/hr/uhag090 (PMC13271787; doi:10.1093/hr/uhag090)
Supplement: Web_Material_uhag090 [file web_material_uhag090.zip › updated_Supplemental (HR-2025-1235.R2).docx]

**Supporting Information**

**H_2_S generated by L-cysteine desulfhydrase enhances** **heat tolerance in tomato via antioxidant capacity and stomatal modulation**

Huihui Fang^1#^, Xiaofang Zhang^1#^, Yunfei Xu^1#^, Wenjia Chen^1^, Kaixin Zheng^1^, Weiling Zhao^1^, Yijie Zang^1^, Yunxiang Zang^1*^

**Supplemental figures**

**
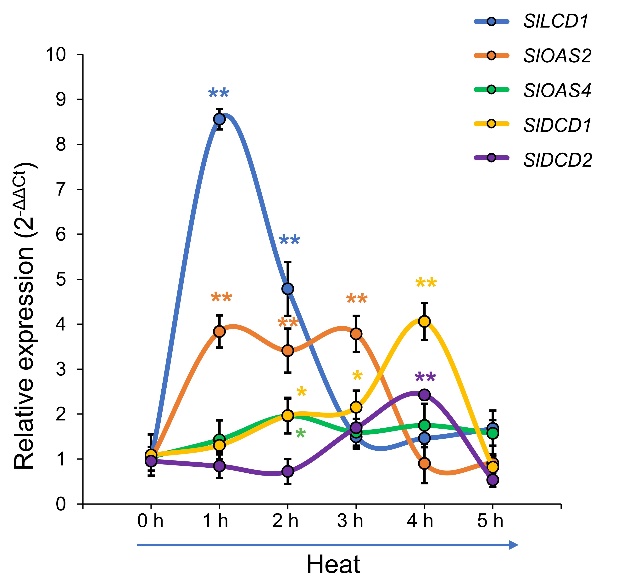
Fig. S1**

**Fig. S1 Dynamic response of key H_2_S-generation-associated genes to heat stress**

The WT tomato plants at the six-true-leaf stage were exposed to 44°C, then the expression levels of key H_2_S-generation-associated genes, including *SlLCD1*, *SlOAS2*, *SlOAS4*, *SlDCD1*, *SlDCD2*, were measured at the indicated time points, 0, 1, 2, 4, and 5 h after heat treatment, respectively. Statistical significance compared to the untreated control (0 h) is indicated as **p* < 0.05 and ***p* < 0.01.

**
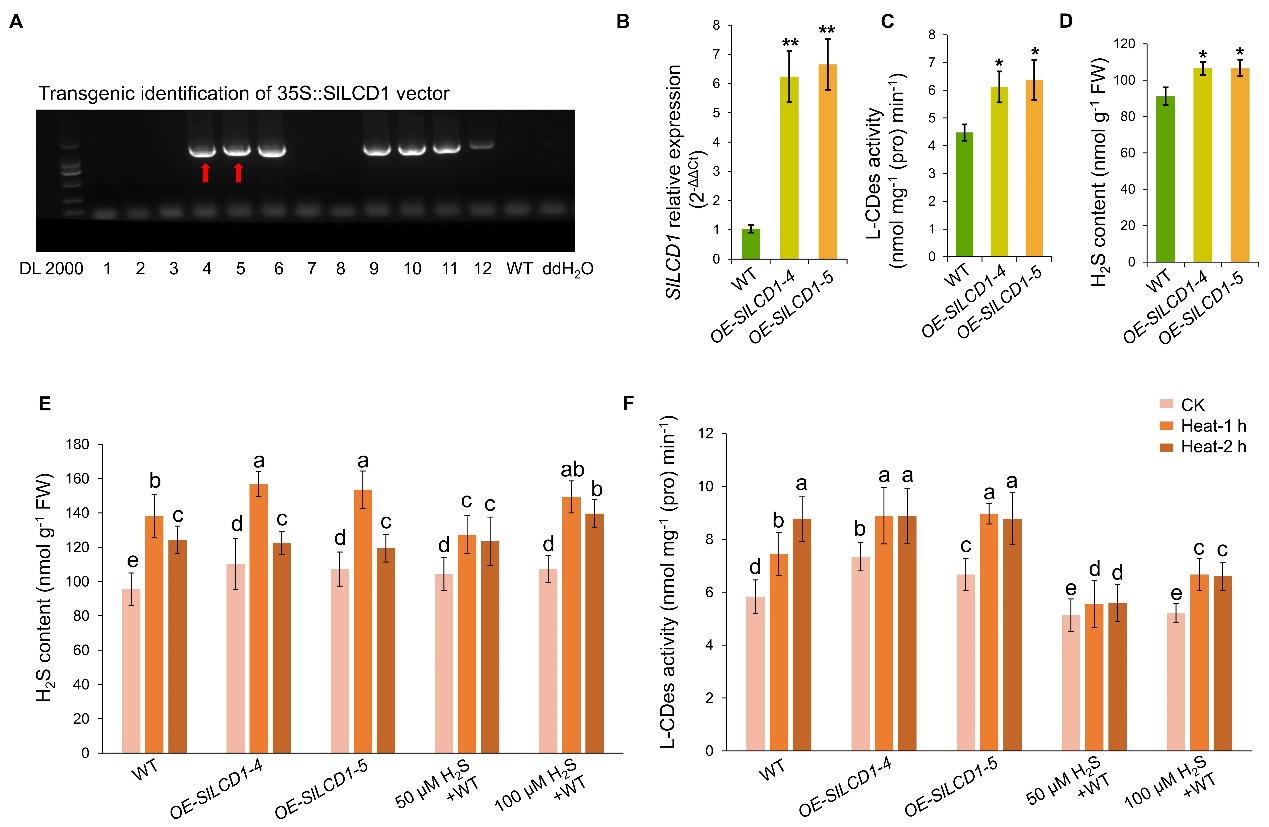
Fig. S2**

**Fig. S2 Overexpression of *SlLCD1* and exogenous H_2_S application modulate endogenous H_2_S production under heat stress.**

**(A)** Molecular identification of transgenic plants carrying the 35S::SlLCD1 vector, and the positive lines were confirmed as *OE-SlLCD1* lines. **(B-D)** The *SlLCD1* expression level (B), L-CDes activity (C), and endogenous H_2_S content (D) in *OE-SlLCD1* lines. **(E-F)** The H_2_S content (E) and L-CDes activity (F) in *OE-SlLCD1* lines and H_2_S-pretreated plants during the early phase of heat stress (0, 1, and 2 h after treatment). The recombinant plasmid for *SlLCD1* overexpression was introduced into tomato via Agrobacterium-mediated genetic transformation. Positive transgenic lines were identified by PCR. Leaves from uniformly grown wild-type (WT) and *OE-SlLCD1* plants were used to measure their H_2_S content and L-CDes activity. Uniformly grown WT plants, *OE-SlLCD1* lines, and H_2_S-pretreated WT plants at the six-true-leaf stage were exposed to 44°C heat stress. Endogenous H_2_S levels and L-CDes activity were analyzed at 1 and 2 h after the onset of heat treatment.

**Fig. S3**

**
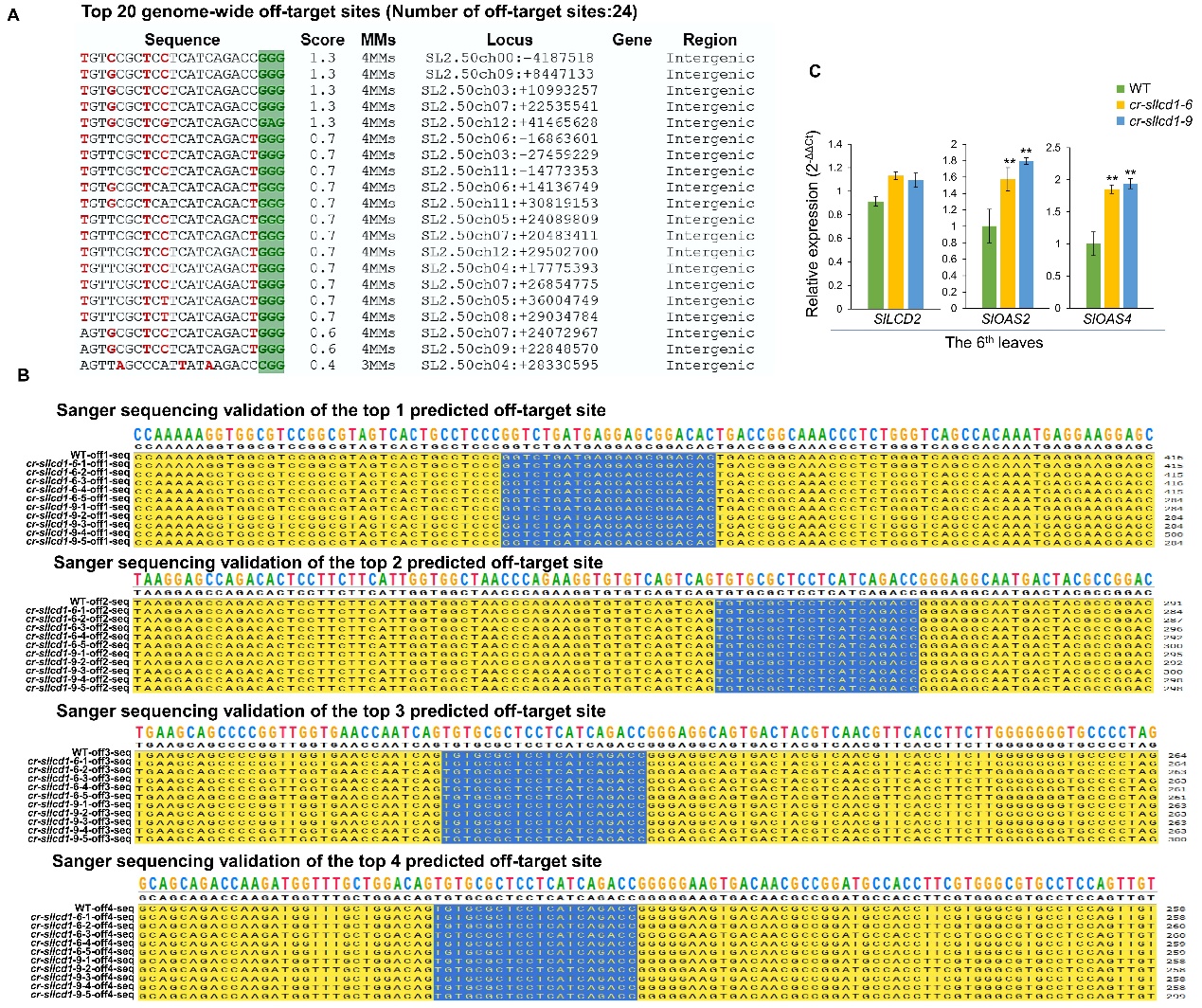
**

**Fig. S3 Assessment of potential off-target effects. (A)** Top 20 genome-wide predicted off-target sites out of 24 candidates. **(B)** PCR validation and Sanger sequencing of the top four potential off-target sites across multiple homozygous mutant lines. Primers are listed in Supplementary Table S2. **(C)** Relative transcript levels of *SlLCD2*, SlOAS2 and SlOAS4 in the 6^th^ leaves of WT and cr-sllcd1 mutants. Leaves were collected from uniformly grown WT and mutant plants at the six‑true‑leaf stage. Asterisks indicate significant differences compared to WT (**p* < 0.05, ***p* < 0.01).

**Fig. S4**

**
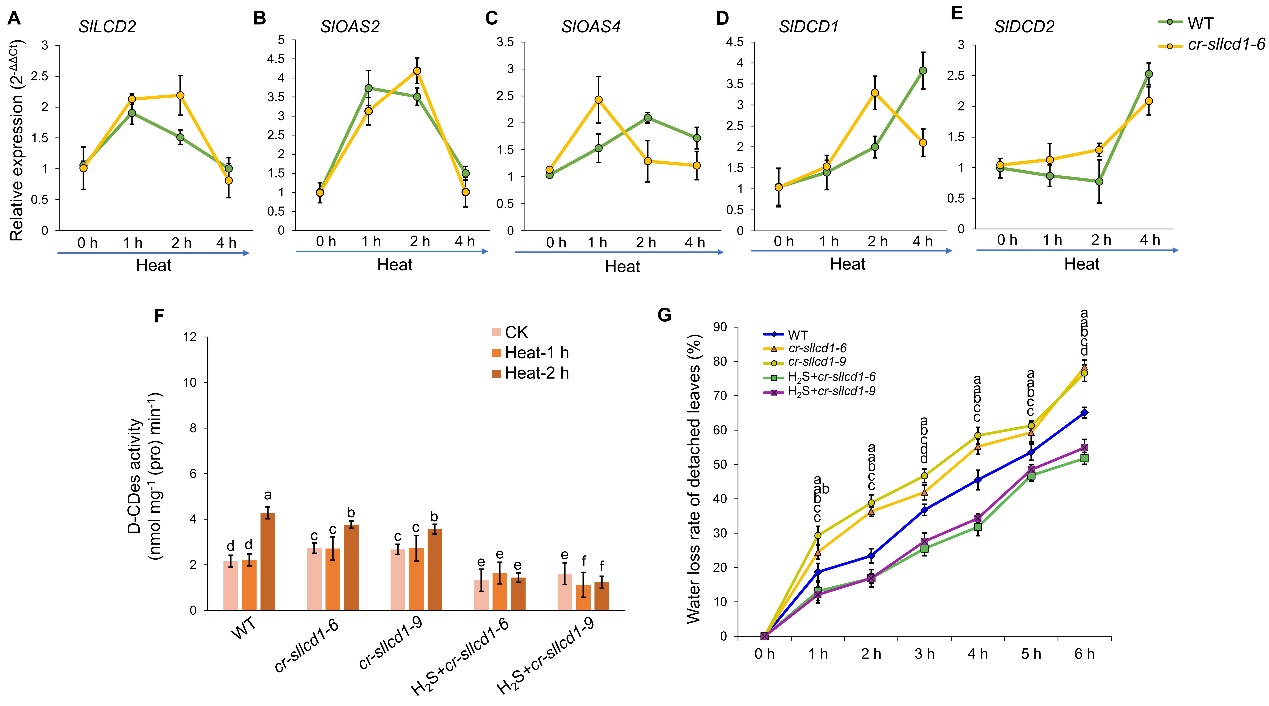
Fig. S4 Heat stress effects on H_2_S metabolism on leaf water loss rate in *cr-sllcd1* mutants.**

**(A-E)** Expression dynamics of *SlLCD2*, SlOAS2, SlOAS4, *SlDCD1*, *SlDCD2* in WT and cr-sllcd1 mutants during heat stress. **(F)** The D-CDes activity in WT, *cr-sllcd1* mutants, and H_2_S-pretreated *cr-sllcd1* mutants at indicated time points under heat stress. **(G)** The water loss rate of detached leaves in WT, *cr-sllcd1* mutants, and H_2_S-pretreated *cr-sllcd1* mutants at indicated time points under heat stress. Uniformly grown plants of WT, *cr-sllcd1-6*, *cr-sllcd1-9*, H_2_S pretreated *cr-sllcd1* mutants (designated H_2_S+*cr-sllcd1-6*, H_2_S+*cr-sllcd1-9*) at the same growth stage (the six-true-leaf stage) were subjected to 44°C heat stress. The gene expression level was detected at 0, 1, 2, 4 h after heat stress. The D-CDes activity were assessed at 1 and 2 h post-treatment. The water loss rate was determined after 5 h of stress. Different letters represent statistically significant differences (*p* < 0.05)

**Methods S1 Determination of detached leaf water loss rate**

The water loss rate of detached leaves was measured by continuous gravimetric monitoring. Immediately after detachment, leaves were trimmed and weighed to record the initial fresh weight (W₀). They were then placed in petri dishes at room temperature, and their fresh weight (Wₜ) was measured hourly. The relative water loss rate was calculated as (W₀ − Wₜ) / W₀ × 100%, representing cumulative water loss relative to the initial weight. Each treatment included three biological replicates, with each replicate consisting of five leaves.

| **Fumigation** | **Species** | **Study** | **reference** |
| --- | --- | --- | --- |
| 50 μM | Arabidopsis | Chromium stress | (Fang et al., 2016; Fang et al., 2017) [1,2] |
| 50 μM | Foxtail millet | Chromium stress | (Fang et al., 2014) [3] |
| 50 μM | Chinese cabbage | Stomatal movement | (Zhang et al., 2025) [4] |
| 50 μM | Arabidopsis | Flowering | (Ma et al., 2024) [5] |
| 50 μM | Arabidopsis | Osmotic stress | (Wang et al., 2022) [6] |
| 75 μM | Arabidopsis | Phosphate starvation | (Liu et al., 2024) [7] |
| 80 μM | Arabidopsis | Stomatal movement | (Jin et al., 2011) [8] |
| 100 μM | Chinese cabbage | Drought stress | (Zhang et al., 2023) [9] |
| 100 μM | Chinese cabbage | Flowering | (Hao et al., 2025) [10] |
| 100 μM | Soybean | Symbiotic nodulation | (Zhang et al., 2025) [11] |
| 100 μM | Arabidopsis | Salinity stress | (Yang et al., 2023) [12] |
| 100 μM | Tomato | Salinity stress | (Ertan et al., 2023; Subba et al., 2025) [13,14] |
| 100 μM | Tomato | Seedling growth | (Ba et al., 2021) [15] |
| 200 μM | Salt-secreting mangrove plant Avicennia marina | Na^+^ and K^+^ fluxes | (Ming-Yue et al., 2022) [16] |
| 900 μM | Tomato | Fruit ripening | (Yao et al., 2018; Chen et al., 2024) [17,18] |

**Table S1 A review of NaHS concentrations used as an exogenous H_2_S donor in treatments.**

**Table S2 All primers used in this study.**

| Primer | Gene ID | Sequence（5’-3’） |  |
| --- | --- | --- | --- |
| OE-SlLCD-F | Solyc01g068160 | tctagaggcgcgcctggtaccATGGAACCGGCGAACGAC | Construction of 35S::SlLCD recombinant plasmid |
| OE-SlLCD-R |  | taatcgcccccgggaagatctGCTTCGGAGGAATTCATCTTACAAGTT |  |
| SlLCD-ID-F |  | ATGGAACCGGCGAACGAC | Transgenic identification of SlLCD-overexpression vector |
| OE-ID-R |  | GGAAATTCGAGCTCCTAGAAC |  |
| U6.26-ID-F |  | TGTCCCAGGATTAGAATGATTAGGC | Transgenic identification of CRISPR vector |
| SlLCD-gRNA-R |  | GGTCTGATGATGGGCGAACT |  |
| GSLCD-CX-F |  | TGTAGCAGAGTCAGTTACCT | Sequencing verification of gene editing |
| GSLCD-CX-R |  | GAAACTCCGCAACAATCTCT |  |
| q*SlACTIN* | Solyc03g078400 | CTCTACATACTTGAGAGGTGCC | RT-qPCR primers |
|  |  | AGACGAGGAGAAAACATCACAA |  |
| q*SlLCD1* | Solyc01g068160 | CATTGTGCGTTTCAGGCTGTTA |  |
|  |  | GGGCAAATGAACAACGATCACA |  |
| q*SlLCD2* | Solyc05g007590 | AGATCGGAGAAGGTGGTAGAT |  |
|  |  | CACTATAATCCCTCGTCCCAATC |  |
| q*SlOAS2* | Solyc01g097930 | AAATAGTGAAGTCCTCCCAAGTT |  |
|  |  | ACAGCTATGGAATGATGAAAATG |  |
| q*SlOAS4* | Solyc01g097930 | AGTTGAGTTAGGCCTAGTGTCATA |  |
|  |  | GTGCTTCCAATAAGTTCCGT |  |
| q*SlDCD1* | Solyc03g098230 | GGGTACGGTTGCTGGTTTGT |  |
|  |  | CTCATCAGTGGTGCTCAAAG |  |
| q*SlDCD2* | Solyc01g008900 | GAAAGATTGCTCAAGAAACGGG |  |
|  |  | GAGCTAGACCAAACATACCGAG |  |

**References**

1. Fang H, Liu Z, Long Y. et al. The Ca^2+^/calmodulin2-binding transcription factor TGA3 elevates LCD expression and H_2_S production to bolster Cr^6+^ tolerance in Arabidopsis. Plant J. 2017;**91**:1038–50.

2. Fang H, Liu Z, Jin Z. et al. An emphasis of hydrogen sulfide-cysteine cycle on enhancing the tolerance to chromium stress in Arabidopsis. Environ Pollut. 2016;**213**:870–7.

3. Fang H, Jing T, Liu Z. et al. Hydrogen sulfide interacts with calcium signaling to enhance the chromium tolerance in *Setaria italica*. Cell Calcium. 2014;**56**:472–81.

4. Zhang J, Zhang L, Han X. et al. H_2_S modulates BrSDH1-1 alternative splicing to induce stomatal closure in Chinese cabbage. Hortic Res. 2025;**12**:uhaf214.

5. Ma T, Xu S, Wang Y. et al. Exogenous hydrogen sulphide promotes plant flowering through the Arabidopsis splicing factor AtU2AF65a. Plant Cell Environ. 2024;**47**:1782–96.

6. Wang Z, Mu Y, Hao X. et al. H_2_S aids osmotic stress resistance by S-sulfhydration of melatonin production-related enzymes in *Arabidopsis thaliana*. Plant Cell Rep. 2022;**41**:365–76.

7. Liu T, Chen H, Luo S. et al. Hydrogen sulphide alleviates root growth inhibition induced by phosphate starvation. Plant Cell Environ. 2024;**47**:5265–79.

8. Jin Z, Shen J, Qiao Z. et al. Hydrogen sulfide improves drought resistance in Arabidopsis thaliana. Biochem Biophys Res Commun. 2011;**414**:481–6.

9. Zhang W, Wang L, Zhang L. et al. H_2_S-mediated balance regulation of stomatal and non-stomatal factors responding to drought stress in Chinese cabbage. Hortic Res. 2023;**10**:uhac284.

10. Hao X, Li W, Cao H. et al. H_2_S promotes flowering in *Brassica rapa* ssp. *pekinensis* by persulfidation of the splicing factor BraATO2. Hortic Res. 2025;**12**:uhaf190.

11. Zhang W, Liu W, Wang K. et al. Persulfidation of host NADPH oxidase RbohB by rhizobial 3-mercaptopyruvate sulfurtransferase maintains redox homeostasis and promotes symbiotic nodulation in soybean. Mol Plant. 2025;**18:**1843–63.

12. Yang L, Yang J, Hou C. et al. Hydrogen sulfide alleviates salt stress through auxin signaling in Arabidopsis. Environ Exp Bot. 2023;**211**:105354.

13. Ertan Y, Melek E, Metin T. et al. Physiological, Morphological and Biochemical Responses of Exogenous Hydrogen Sulfide in Salt-Stressed Tomato Seedlings. Sustainability. 2023;**15**:1098.

14. Subba R, Mukherjee S, Mathur P. Exogenous H_2_S regulates growth, and antioxidative defense in tomato seedings in a concentration dependent manner under salt stress. Protoplasma. 2025.

15. Ba Y, Zhai J, Yan J. et al. H_2_S improves growth of tomato seedlings involving the MAPK signaling. Sci Hortic. 2021;**288**:110366.

16. Ming-Yue W, Huan L, Lu-Dan Z. et al. Exogenous hydrogen sulfide mediates Na^+^ and K^+^ fluxes of salt gland in salt-secreting mangrove plant Avicennia marina. Tree Physiol. 2022;**42**:1812–1826.

17. Yao G, Wei Z, Li T. et al. Modulation of enhanced antioxidant activity by hydrogen sulfide antagonization of ethylene in tomato fruit ripening. J Agric Food Chem. 2018;**66**:10380–7.

18. Chen XC, Man X, Huang DH. et al. Hydrogen sulfide inhibits tomato fruit ripening by restraining fruit softening and ethylene production. LWT. 2024;**200**:116210.
