# Supplementary figures and images for: H2S generated by L-cysteine desulfhydrase (SlLCD1) enhances heat tolerance in tomato via antioxidant capacity and stomatal modulation
Source: Hortic Res. 2026 Mar 9;13(7):uhag090. doi: 10.1093/hr/uhag090 (PMC13271787; doi:10.1093/hr/uhag090)

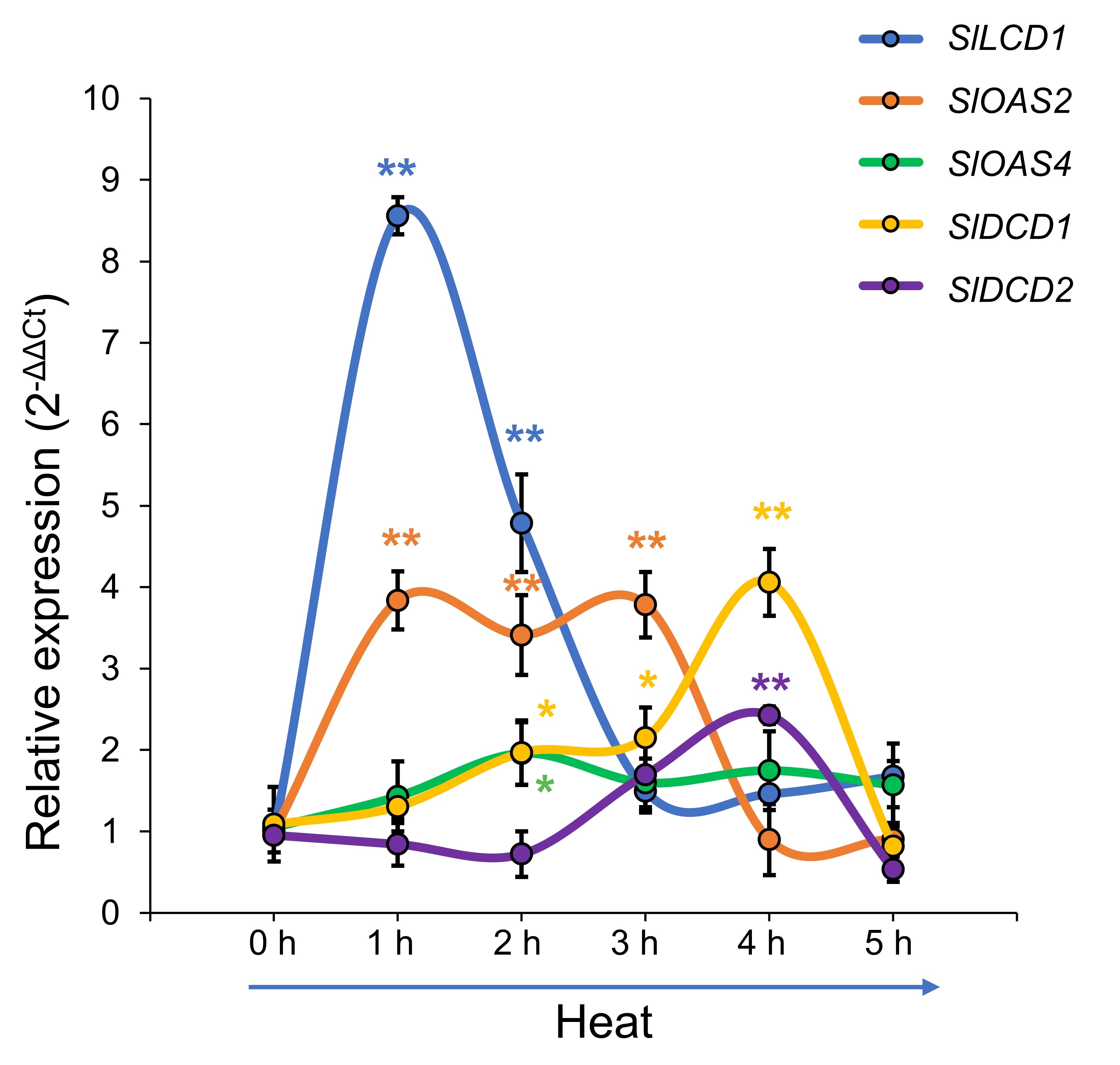

Supplement: Web_Material_uhag090 [file web_material_uhag090.zip › Fig.S1.jpg]

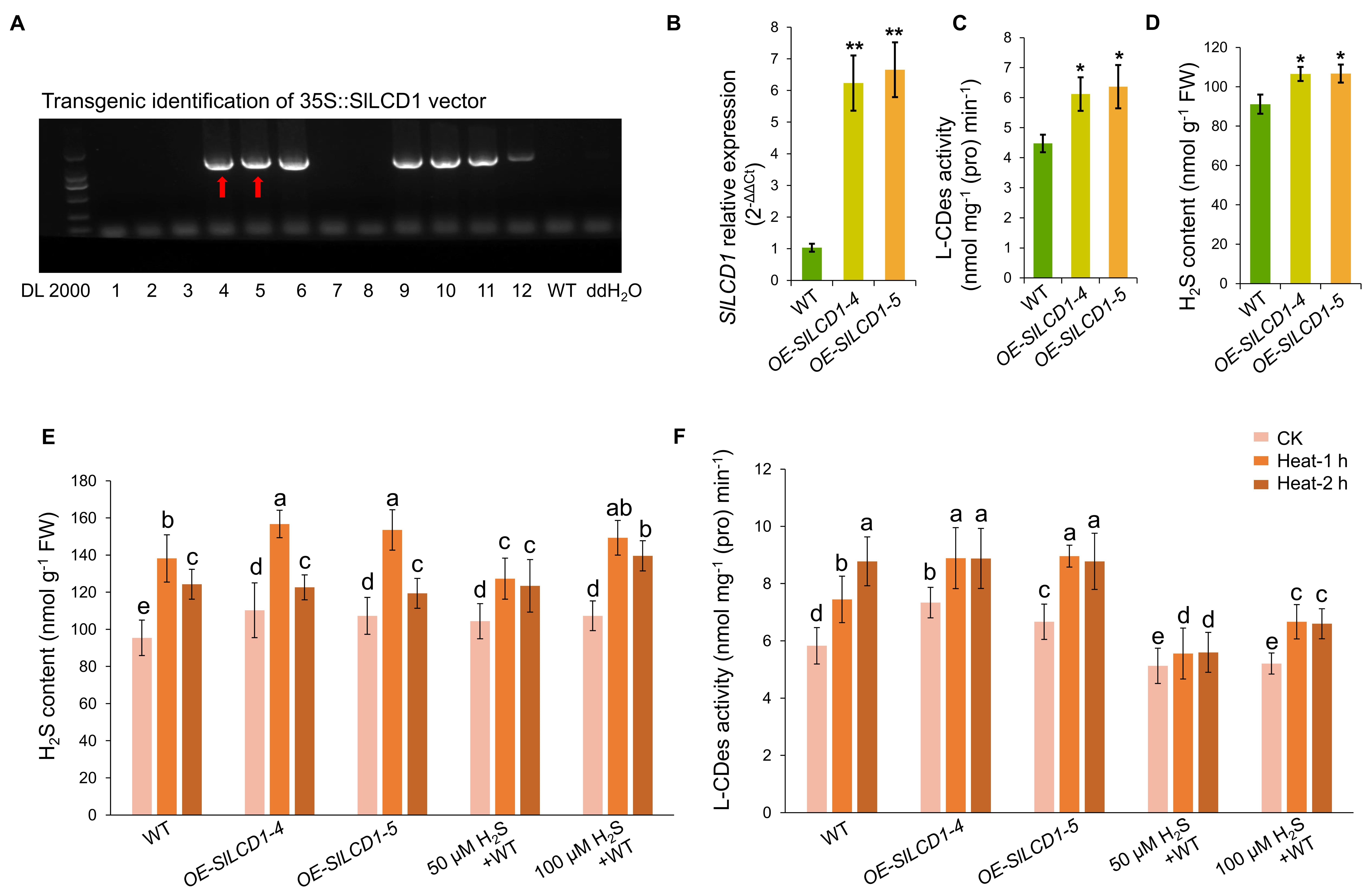

Supplement: Web_Material_uhag090 [file web_material_uhag090.zip › Fig.S2.jpg]

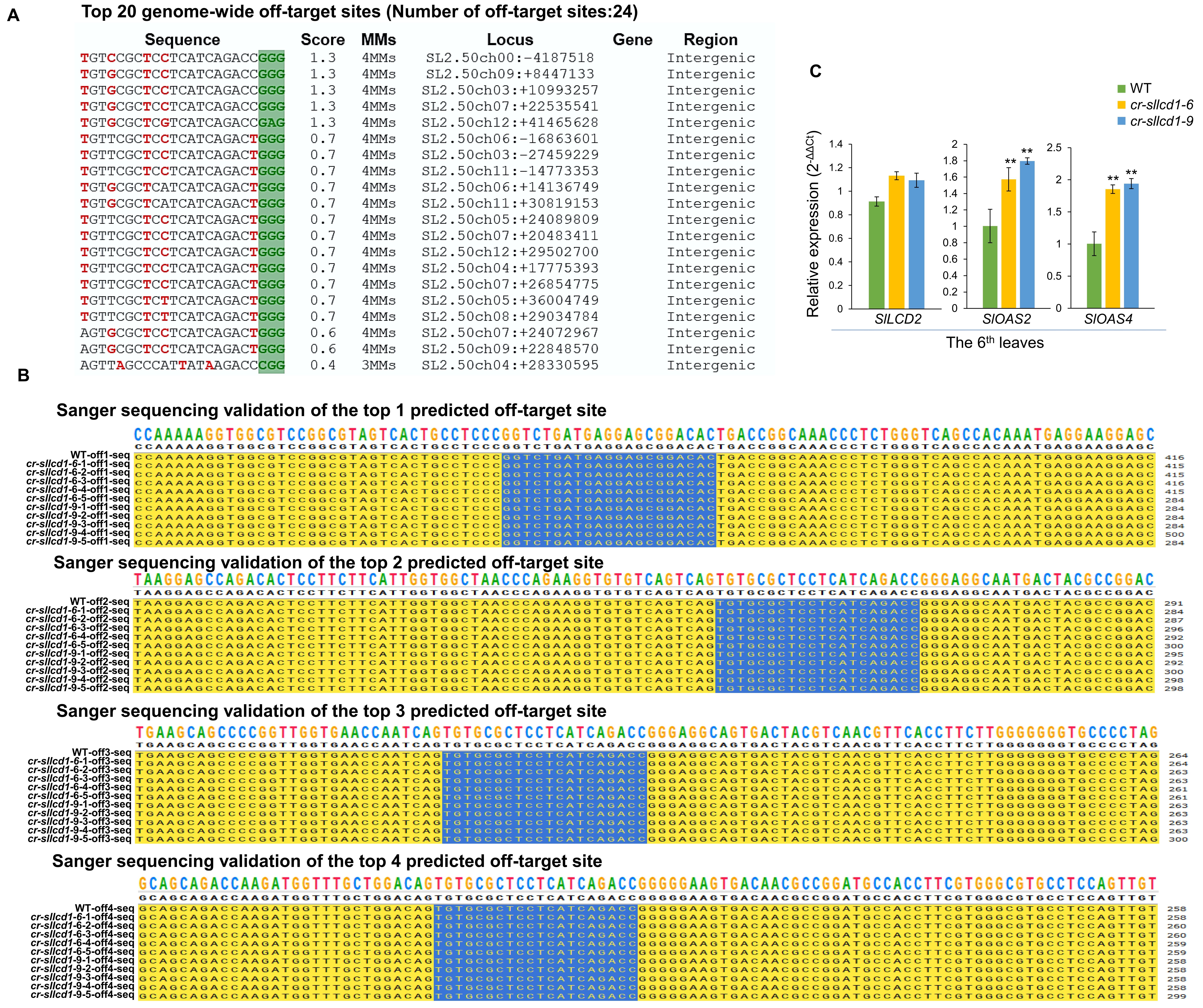

Supplement: Web_Material_uhag090 [file web_material_uhag090.zip › Fig.S3.jpg]

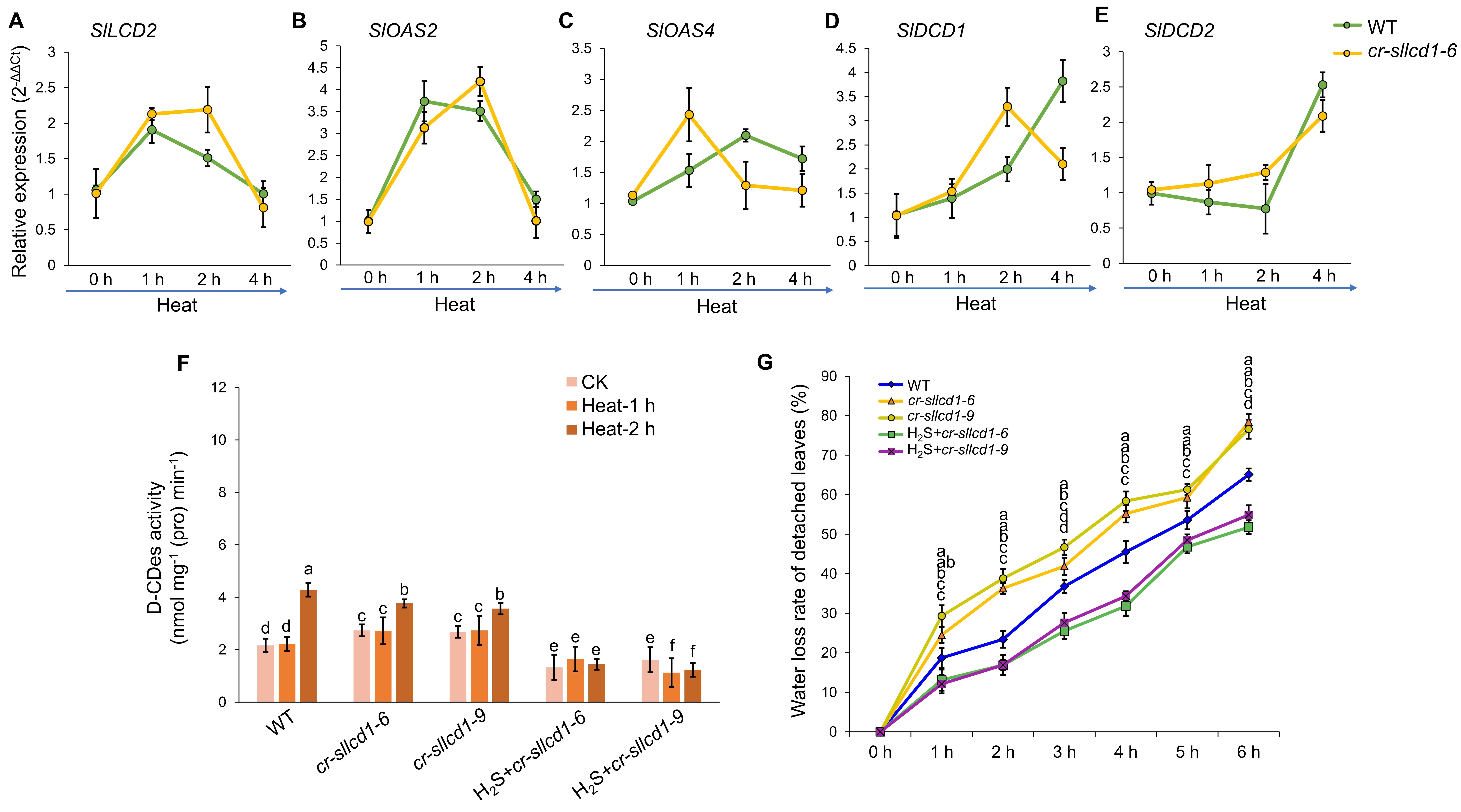

Supplement: Web_Material_uhag090 [file web_material_uhag090.zip › updated supplemental Fig.S4 (HR-2025-1235.R2).jpg]
